# Association of reduced inner retinal thicknesses with chronic kidney disease

**DOI:** 10.1186/s12882-019-1679-1

**Published:** 2020-01-31

**Authors:** Euan N. Paterson, Meera L. Ravindran, Kayleigh Griffiths, Claire A. Le Velly, Chris C. Cardwell, Rachel V. McCarter, Patrick Nicol, Jay K. Chhablani, Mohammed Abdul Rasheed, Kiran Kumar Vupparaboina, Thomas J. MacGillivray, Mark Harbinson, Alexander P. Maxwell, Ruth E. Hogg, Gareth J. McKay

**Affiliations:** 1 Centre for Public Health, Queen’s University Belfast, Block B, Royal Hospital, Grosvenor Road, Belfast, Northern Ireland BT12 6BA; 2 Department of Ophthalmology, University of Pittsburgh Medical Center, Pittsburgh, USA; 3 L.V. Prasad Eye Institute, Hyderabad, India; 4 Centre for Clinical Brain Sciences, University of Edinburgh, Edinburgh, UK; 5 Centre for Medical Education, Queen’s University Belfast, Belfast, Northern Ireland

**Keywords:** Renal function, Chronic kidney disease, Retina, Retinal microvasculature, Choroid, Retinal thickness, Choroidal volume, Choroidal vascularity index

## Abstract

**Background:**

Tissue derived biomarkers may offer utility as indicators of accumulated damage. Reduced thickness of retinal neuronal tissue and the vascular choroid have previously been associated with vascular damage and diabetes. We evaluated associations between retinal thickness, retinal microvascular and choroidal measures, and renal function in a population with a high burden of comorbidity.

**Methods:**

Participants were recruited from nuclear cardiology or renal medicine clinics. Retinal and choroidal thickness were measured from spectral-domain optical coherence tomograms. Retinal microvascular parameters were assessed from digital fundus photographs using a semi-automated software package. Main Outcome Measure: Chronic kidney disease (CKD) categorised as: CKD stages 1–2, eGFR ≥60 ml/min/1.73m^2^; CKD stage 3, eGFR 30–59 ml/min/1.73m^2^, and CKD stages 4–5, eGFR ≤29 ml/min/1.73m^2^.

**Results:**

Participants (*n* = 241) had a mean age of 65 years and a mean eGFR of 66.9 ml/min/1.73m^2^. Thirty–nine % of the cohort had diabetes and 27% were using diuretics. Thinning of the inner retina and changes to its microvascular blood supply were associated with lower eGFR and CKD stages 4 and 5, while no associations were found between the outer retinal layers or their choroidal blood supply and CKD of any stage. These associations remained following adjustment for age, mean arterial blood pressure, diabetes status, low-density lipoprotein, body mass index, and sex.

**Conclusions:**

Inner retinal thinning and retinal microvascular variation is associated with advanced CKD (stages 4 & 5) independent of important confounding factors, but not with earlier stage CKD (stage 3) and, therefore, its utility as a biomarker for early CKD is not supported in this study.

## Background

Chronic kidney disease (CKD) is a major global health concern estimated to affect between 3 and 18% of the population [1, 2], resulting in a substantial economic burden [3–5] and reduced quality of life [6, 7]. Incidence and prevalence rates for CKD are predicted to increase significantly over the coming decades given rising obesity rates and ageing populations [8]. As such, non-invasive, early-stage detection methods would offer significant clinical utility for identification of persons with CKD so that targeted interventions could be offered to reduce renal decline [9].

Despite the availability of several indicators of renal function and damage, such as serum creatinine, cystatin C, and proteinuria, the ability to identify those at greatest risk of future renal decline is limited [10]. The use of a variety of circulating and genetic biomarkers has offered improved CKD detection and risk prediction [11]. Tissue derived biomarkers provide utility as indicators of accumulated damage, such as damage to the vasculature resulting from non-traditional CKD risk factors [12–16], however they are typically less amenable to non-invasive assessment [17].

Improved retinal imaging modalities and analysis software has resulted in reported associations between retinal microvascular variation and renal function, independent of hypertension and diabetes [18–23]. Such associations may reflect systemic vascular effects and reno-vascular damage [24]. Indeed, overlapping physiology between the cells of the renal and ocular microcirculation, including specialised cell types such as retinal pericytes and renal mesangial cells, highlight the potential for similar pathological pathways in both the eye and the kidney [24]. More recently, evaluation of retinal thickness through non-invasive optical coherence tomography (OCT) has been considered as a potential biomarker for kidney damage [25].

The retina consists of multiple neuronal layers which can be imaged non-invasively using OCT. Variability in the thickness of the retinal layers has been associated with several chronic conditions, including diabetes mellitus [26–28] and hypertension [29], both major contributory risk factors for CKD. Recent associations between CKD and thinning of the retinal and adjacent vascular choroidal layers were reported using OCT in a population without diabetes or cardiovascular disease (CVD) [25]. However, the choroidal vasculature supplies only the outer layers of the retina, with the inner layers supplied by the retinal microvasculature. A study examining the concordance between differences in the thickness of the individual layers of the retina and their specific vascular supply in association with CKD is warranted. The aim of this study was to evaluate retinal thickness and microvascular measures in association with renal function in a population with a high burden of comorbidity, independent of important confounding factors.

## Methods

A cross-sectional analysis of participants who attended the nuclear cardiology and renal medicine clinics at the Royal Victoria and Belfast City Hospitals was undertaken between September 2015 and March 2017. Patients attending the nuclear cardiology clinic have cardiovascular risk factors or cardiovascular disease, and may be at risk of CKD; as such, they form a population where non-invasive assessment regarding CKD may be of value. Inclusion criteria were participant age ≥ 18 years and ability to provide informed consent. Ethics Committee approval was obtained from the Office for Research Ethics Committees Northern Ireland (Study ID 14/NI/1132) and conformed to the guidelines of the 1975 Declaration of Helsinki.

### Assessment of CKD status

Serum creatinine values were obtained from NHS laboratory measurements taken through routine clinical assessment and estimated glomerular filtration rate (eGFR) was calculated using the CKD-EPI equation as a measure of renal function [30]. CKD was categorised as follows: CKD stages 1–2, eGFR ≥60 ml/min/1.73m^2^; CKD stage 3, eGFR 30–59 ml/min/1.73m^2^, and CKD stages 4–5, eGFR ≤29 ml/min/1.73m^2^.

### Image acquisition

Images were captured using spectral domain optical coherence tomography (SD-OCT) (SPECTRALIS® HRA + OCT imaging platform, Heidelberg Engineering Ltd. Hemel Hempstead, Hertfordshire, United Kingdom) following pupil dilation by administration of a single drop of 1% tropicamide. Posterior pole scans were acquired in high-speed mode employing 768 A-scans per B-scan, over a 9.2 × 7.6 mm (30°× 25°) area, with the fovea remaining central. Sixty-one horizontal B-scans were acquired using automatic real-time tracking (ART) set to 9, with a gap of 120 μm between B-scans. Scans with significant artefacts or substantial mirror edge were discarded. The choroid was imaged through SD-OCT enhanced depth imaging (EDI) also using the SPECTRALIS® HRA + OCT imaging platform (Heidelberg Engineering Inc.) in high-speed mode with a 30°× 25° EDI volume scan, for 19 sections, with ART set to 9.

### Image processing and segmentation

Fovea detection and automated algorithmic segmentation of the retina into constituent layers for retinal thickness assessment was performed using the Heidelberg Eye Explorer (HEYEX, version 1.9.17.0.). Constituent layers that comprised the overall retinal thickness included the inner retinal layer (IRL), outer retinal layer (ORL), nerve fibre layer (NFL), ganglion cell layer (GCL), inner plexiform layer (IPL), inner nuclear layer (INL), outer plexiform layer (OPL), Henle’s nerve fibre layer and outer nuclear layer (HNFL-ONL), and retinal pigment epithelium (RPE) in accordance with the International Nomenclature for Optical Coherence Tomography (IN*OCT) consensus [31].

**Fig. 1:**
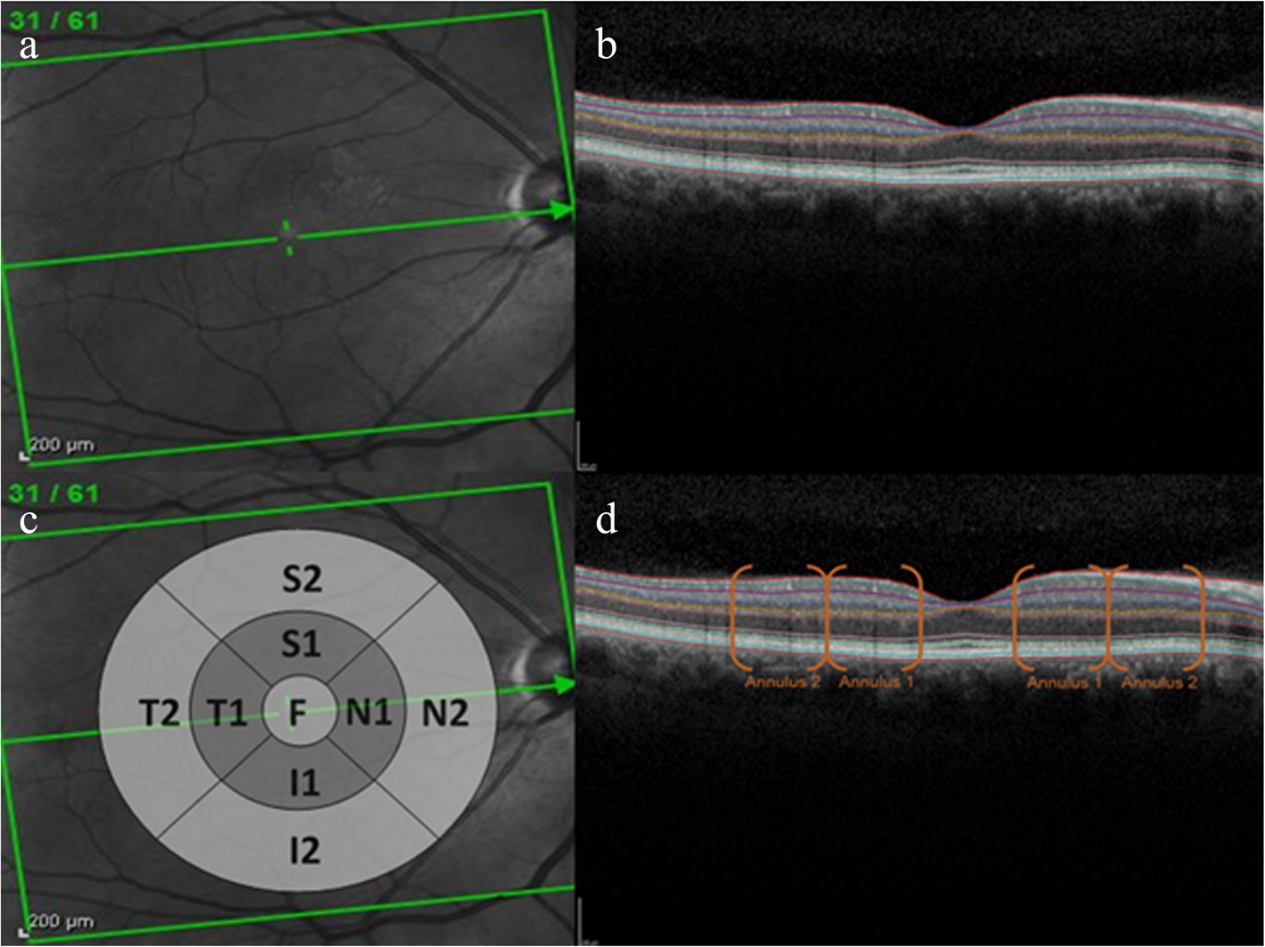
Retinal images and grid positions: **a** shows an image of the retina *en face*. **b** shows a cross-sectional image with differentiation of the retinal layers using the HAYEX software. The cross-section of the retina represents the layers directly behind the green line bisecting the *en face* image in panel A. The cross hairs in the left hand panel indicate the location of the foveal dip which can be seen as a depression in the centre of the image in panel B. **c** indicates the approximate position and size of the ETDRS grid used for reporting retinal thickness. Segment F is centred over the fovea. The annulus proximal to the fovea (annulus 1) comprises segments; S1 = superior 1; N1 = nasal 1; I1 = inferior 1; T1 = temporal 1. The annulus distal to the fovea comprises segments; S2 = superior 2; N2 = nasal 2; I2 = inferior 2; T2 = temporal 2. **d** highlights the locations where the ETDRS grid segments bisect the retinal image shown in panel B

Scans were examined and segmentation errors amended by graders masked to all clinical information to avoid any potential bias. For each scan, the foveal centre was identified as the frame including the brightest foveal reflex and the thickness of the individual layers was recorded in microns at the point at which the software caliper intersected the foveal reflex. Examples of *en face* and cross-sectional retinal images are provided with Early Treatment Diabetic Retinopathy Study (ETDRS) grid locations noted (Fig. 1). Thickness of the retinal layers were measured within standardised segments on an ETDRS grid centred on the fovea. These segments describe four quadrants (inferior [I], superior [S], temporal [T], and nasal [N]) for each of two annuli (annulus 1, proximal to the fovea, and annulus 2, distal to the fovea), in addition to a central/foveal segment (F). Overall retinal thickness was measured as the vertical distance between Bruch’s membrane and the vitreoretinal interface. Intraclass correlation coefficients were used to measure the intragrader reliability of retinal thicknesses, assessed in 10 retinal images by two trained graders. Mean intraclass correlation coefficient for each ETDRS segment was calculated as 0.97 (F), 0.99 (N1), 0.95 (N2), 0.99 (S1), 0.98 (S2), 0.99 (T1), 0.99 (T2), 0.99 (I1), 0.95 (I2), indicating excellent inter-operator agreement. For choroidal measures, the choroidal images were binarised so that luminal space was represented by dark pixels and the choroidal stroma was represented by light pixels. Choroidal vascularity index (CVI) was calculated using a previously reported algorithm [32].

### Retinal microvascular assessment

Fundus photographs were captured using a 45° retinal fundus camera (Canon CR-DGi; Canon, Tokyo, Japan). Poor quality images were excluded. Retinal microvascular parameters (central retinal arteriolar equivalent [CRAE], central retinal venular equivalent [CRVE], arteriovenous ratio [AVR], and fractal dimensions) were measured from digital retinal fundus photographs using VAMPIRE version 3.1.4 (Universities of Dundee and Edinburgh, Scotland). Retinal microvascular parameters were measured within an annular zone from 1.0 to 1.5 times the optic disc diameter from the centre of the optic disc.

### Statistical analysis

All statistical analyses were performed using IBM SPSS statistics version 23.0 (IBM Corp., Armonk, NY). The difference in mean retinal thickness between CKD categories was assessed using ANOVA and the Student-Newman-Keuls test was used to identify differences between CKD categories. Multinomial logistic regression, with CKD in categories (grouped as stages 1 to 2, 3 and 4 to 5) as the outcome, was used to test associations between retinal thickness and CKD category, and associations between retinal microvascular parameters, CVI, and choroidal volume and CKD categories. Multivariable linear regression was used to assess associations between retinal thickness and eGFR, with the latter as the outcome measure. Regression models were adjusted for age, mean arterial blood pressure (MABP), diabetes status, low-density lipoprotein (LDL), body mass index (BMI), and sex. Associations detected in both eyes were considered significant only if detected bilaterally in both left and right eyes.

The data generated during the current study are available from the corresponding author on reasonable request.

## Results

Consent for OCT imaging was provided by 241 of the 252 study participants. Of these, 18 participant’s images were of insufficient quality for grading leaving 223 participants with measurable images. The mean age of the population was 65 years (standard deviation [SD] 9.5), 44% were female and the mean BMI was 31.0 kg/m^2^ (SD 6.4) (Table 1). The mean systolic and diastolic blood pressures were 136 mmHg (SD 22) and 81 mmHg (SD 13) respectively. A high disease burden was evident with 35% prescribed antihypertensive medication and 39% having diabetes. Mean HbA1c was 53 mmol/mol (SD 18), with 29% of participants taking hypoglycaemic medications. In participants with diabetes, duration of diabetes was less than 5 years in 38%, 5–10 years in 26%, and > 10 years in 32%. Approximately 8% had a history of cerebrovascular accident. Mean total cholesterol (3.9 mmol/L, SD 1.2), and LDL cholesterol (2.3 mmol/L, SD 1.0) were within the healthy range but 70% of the recruited participants were prescribed statins. Mean eGFR was 67 ml/min/1.73m^2^ (SD 25) with 27% of the population using diuretics. Smoking status identified 15% of participants as current smokers and 49% as having never smoked.

**Table 1:** Participant characteristics

Variable	Population value
Age, years, mean (SD)	65.3 (9.5)
Female, n (%)	106 (44)
eGFR, ml/min/1.73m^2^, mean (SD)	67 (25)
Weight, Kg, mean (SD)	89.0 (20.1)
BMI, kg/m^2^, mean (SD)	31.0 (6.4)
Hypertension, n (%)	157 (65)
SBP, mmHg, mean (SD)	136 (22)
DBP, mmHg, mean (SD)	81 (13)
MABP (1/3 SBP + 2/3 DBP), mmHg, mean (SD)	99 (13)
HbA1c mean, mmol/mol, (SD)	53 (18)
Total Cholesterol, mean, mmol/L (SD)	3.92 (1.18)
HDL, mean, mmol/L (SD)	1.33 (0.45)
LDL, mean, mmol/L (SD)	2.28 (0.97)
Troponin, mean, mmol/L (SD)	17.8 (28.9)
CRP, mean, mmol/L (SD)	9.5 (35.6)
Pack Years, mean (SD)	39.8 (35.0)
CVD, n (%)	100 (41.5)
Diabetes mellitus, n (%)	93 (39)
Type 1, n (%)	13 (5)
Type 2, n (%)	80 (33)
Insulin, n (%)	29 (31)
Previous CVA, n (%)	19 (8)
Antihypertensives, n (%)	83 (35)
Hypoglycaemics, n (%)	68 (29)
Statins, n (%)	164 (69)
Diuretics, n (%)	63 (27)
NSAIDs, n (%)	12 (5)
Vitamin supplements, n (%)	48 (20)
Rheumatoid arthritis, n (%)	10 (9)
Hypertension treatment, n (%)	
None	78 (33)
< 5 years	54 (23)
5–10 years	38 (16)
> 10 years	64 (27)
Smoking, n (%)	
Never	119 (49)
Ex	87 (36)
Current	35 (15)
Alcohol, n (%)	
none	134 (56)
1–5 units	38 (16)
6–10 units	28 (12)
15–20 units	25 (10)
> 25 units	12 (5)
QRISK2 score, n (%)	
1	38 (34)
2	35 (31)
3	38 (34)

*SD* Standard deviation, *eGFR* estimated glomerular filtration rate, *SBP* Systolic blood pressure, *DBP* Diastolic blood pressure, *MABP* Mean arterial blood pressure, *HbA1c* Glycated haemoglobin A1c, *HDL* High density lipoprotein, *LDL* Low density lipoprotein, *CRP* C reactive protein, *CVD* Cardiovascular disease, *CVA* Cerebrovascular accident, *NSAIDs* Non-steroidal anti-inflammatory drugs

### Retinal thickness

Mean retinal thickness values are presented according to the ETDRS grid configuration (Fig. 1, Table 2). A descriptive map of individual retinal layers for reference can be found in the consensus statement of the INOCT group [31]. Mean retinal thickness was significantly lower in the nasal, superior and annulus 1 inferior segments in participants with CKD stages 4–5. There were no significant differences in retinal thickness between CKD categories for the central/foveal segment, nor for any of the annulus distal segments (data not shown).

**Table 2:** Mean retinal, inner retinal and outer retinal thickness values for CKD stages 1–2, 3, and 4–5

ETDRS segment	Right eye				Left eye			
	CKD stages 1–2	CKD stage 3	CKD stage 4–5	ANOVA	CKD stages 1–2	CKD stage 3	CKD stage 4–5	ANOVA
	Mean thickness μm (SD)	Mean thickness μm (SD)	Mean thickness μm (SD)	p	Mean thickness μm (SD)	Mean thickness μm (SD)	Mean thickness μm (SD)	p
Full retinal thickness								
N1	342 (17) ^A^	339 (21) ^A^	324 (27) ^B^	< 0.001*	343 (22) ^A^	341 (26) ^A^	327 (28) ^B^	0.004*
S1	337 (18) ^A^	336 (20) ^A^	320 (26) ^B^	< 0.001*	340 (20) ^A^	336 (22) ^A^	321 (28) ^B^	< 0.001*
T1	326 (17)	324 (16)	317 (48)	0.14	328 (19) ^A^	325 (19) ^A^	313 (38) ^B^	0.005*
I1	336 (17) ^A^	332 (19) ^A^	319 (28) ^B^	< 0.001*	337 (21) ^A^	333 (19) ^A^	318 (27) ^B^	< 0.001*
Inner Retinal Layer								
N1	261 (17) ^A^	258 (19) ^A^	244 (26) ^B^	< 0.001*	262 (21) ^A^	257 (19) ^B^	248 (30) ^B^	0.01*
S1	257 (17) ^A^	256 (19) ^A^	241 (25) ^B^	< 0.001*	260 (20) ^A^	255 (19) ^A^	243 (30) ^B^	0.001*
T1	246 (16)	244 (15)	238 (48)	0.19	248 (18) ^A^	245 (18) ^A^	235 (38) ^B^	0.01*
I1	257 (17) ^A^	253 (18) ^A^	240 (28) ^B^	< 0.001*	258 (20) ^A^	254 (16) ^A^	241 (27) ^B^	< 0.001*
Outer Retinal Layer								
N1	81 (3)	81 (4)	80 (3)	0.47	81 (3)	83 (11)	80 (3)	0.06
S1	80 (3)	80 (4)	79 (3)	0.45	80 (3)	81 (7)	79 (3)	0.08
T1	80 (3)	80 (4)	79 (3)	0.29	80 (3)	80 (3)	79 (3)	0.35
I1	79 (3)	79 (4)	79 (4)	0.91	79 (3)	80 (6)	78 (4)	0.15

*ETDRS* Early Treatment Diabetic Retinopathy Study, *OR* Odds ratio, *SD* Standard deviation, *CI* Confidence interval, *F* Fovea, *S1* Superior segment 1, *N1* Nasal segment 1, *I1* Inferior segment 1, *T1* Temporal segment 1. ^A^, ^B^ and ^C^ indicate groups which are significantly different from each other according to SNK test

Odds ratios (OR)s and 95% confidence intervals (CI) were estimated for CKD per μm change in retinal thickness for the full, inner and outer retina according to the ETDRS segments of annulus 1 with adjustment for age, MABP, diabetes, LDL, BMI, and sex (Table 3). Stages 1–2 (defined as eGFR ≥60 ml/min/1.73m^2^) were used as a reference category. Retinal thickness was not associated with CKD stage 3 in any ETDRS segments. For instance, the odds ratio for having CKD stage 3 compared with 1–2 was 1.00 (0.97, 1.02) per μm increase in N1 full retinal thickness. A thicker retina was negatively associated with CKD stages 4–5 for the nasal, superior and inferior annulus 1 segments. For example, the odds ratio for having CKD stage 4–5 compared with 1–2 was 0.97 (0.94, 0.99) per μm increase in N1 full retinal thickness. ORs for the full list of retinal layers by ETDRS segment for both unadjusted and adjusted analyses are provided in Additional file 1: Table S1. Retinal thickness was also negatively associated with CKD stages 4–5 in the temporal segment of annulus 2, but there was no significant association between full retinal thickness and CKD stages in any other segments. Retinal thickness was not significantly associated with CKD stages 4–5 in any other segments in the adjusted models.

**Table 3:** Odds ratios from multinomial logistic regression models for CKD stage 3 and stages 4–5 per μm increase in thickness of the full retina, inner retina and outer retina

ETDRS segment	Right eye						Left eye					
	CKD stage 3 (vs. stage 1–2)			CKD stage 4–5 (vs. stage 1–2)			CKD stage 3 (vs. stage 1–2)			CKD stage 4–5 (vs. stage 1–2)		
	OR	95% CI	p	OR	95% CI	p	OR	95% CI	p	OR	95% CI	p
Full retinal thickness												
N1	1.00	(0.97, 1.02)	0.89	0.97	(0.94, 0.99)	0.02*	1.00	(0.98, 1.01)	0.70	0.98	(0.95, 1.00)	0.03*
S1	1.00	(0.98, 1.03)	0.76	0.97	(0.94, 0.99)	0.01*	1.00	(0.98, 1.01)	0.69	0.97	(0.95, 0.99)	0.01*
T1	1.00	(0.98, 1.02)	0.80	0.99	(0.97, 1.01)	0.33	1.00	(0.98, 1.02)	0.83	0.98	(0.96, 1.00)	0.08
I1	1.00	(0.97, 1.02)	0.87	0.97	(0.95, 1.00)	0.02*	1.00	(0.98, 1.01)	0.73	0.97	(0.94, 0.99)	0.01*
Inner Retinal Layer												
N1	1.00	(0.97, 1.02)	0.70	0.97	(0.94, 0.99)	0.01*	0.99	(0.97, 1.01)	0.32	0.98	(0.95, 1.00)	0.03*
S1	1.00	(0.98, 1.03)	0.87	0.97	(0.94, 0.99)	0.01*	0.99	(0.98, 1.01)	0.47	0.97	(0.95, 0.99)	0.01*
T1	1.00	(0.98, 1.02)	0.92	0.99	(0.96, 1.01)	0.34	1.00	(0.98, 1.01)	0.71	0.98	(0.96, 1.00)	0.09
I1	1.00	(0.97, 1.02)	0.80	0.97	(0.94, 0.99)	0.02*	0.99	(0.97, 1.01)	0.47	0.97	(0.94, 0.99)	0.01*
Outer Retinal Layer												
N1	1.08	(0.96, 1.22)	0.22	1.01	(0.86, 1.18)	0.93	1.12	(0.99, 1.28)	0.08	1.00	(0.85, 1.17)	0.96
S1	1.07	(0.93, 1.22)	0.34	1.00	(0.84, 1.18)	0.96	1.08	(0.96, 1.21)	0.20	0.97	(0.83, 1.13)	0.68
T1	1.10	(0.96, 1.25)	0.19	1.01	(0.84, 1.20)	0.94	1.08	(0.93, 1.26)	0.31	0.99	(0.82, 1.18)	0.90
I1	1.05	(0.92, 1.21)	0.46	1.05	(0.89, 1.25)	0.54	1.09	(0.96, 1.23)	0.18	1.02	(0.88, 1.18)	0.82

*ETDRS* Early Treatment Diabetic Retinopathy Study, *OR* Odds ratio, *SD* Standard deviation, *CI* Confidence interval, *F* Fovea, *S1* Superior segment 1, *N1* Nasal segment 1, *I1* Inferior segment 1, *T1* Temporal segment 1.*significant values. Adjustment for age, mean arterial blood pressure, diabetes status, low-density lipoprotein, body mass index, and sex

In analyses of the inner and outer retinal layers, similar patterns of association were observed between CKD stages 4–5 and the inner retinal layer (Table 3), i.e. a thicker inner retina in the proximal annulus was significantly associated with reduced risk of CKD stage 4–5. Outer retinal thickness was not significantly associated with CKD in adjusted analyses.

### Associations between retinal thickness and retinal microvascular supply

ORs for CKD stages per μm change in retinal thickness are presented for layers primarily supplied by the retinal microvasculature (NFL, GCL, IPL, and INL) for the annulus 1 ETDRS segments in Table 4 with adjustment for age, MABP, diabetes, LDL, BMI, and sex. Greater IPL thickness was associated with reduced risk of CKD stage 3–4 in all inner ETDRS segments and GCL thickness was associated with reduced risk of CKD stage 3–4 in segments T1 and I1. For example, per μm increase in thickness of the IPL in segment S1 of the right eye, the odds ratio for CKD stage 4–5, was 0.89 times that for stage 1–2 (OR 0.89, 95% CI 0.80, 0.99). The associations detected between inner and complete retinal thickness and CKD arose from variation detected in those layers supported by the retinal microvasculature. Specifically, in models adjusted for age, MABP, diabetes status, LDL, BMI, and sex, greater thickness of the GCL (segments T1 and I1) and IPL (all segments of annulus 1 and segment F) were associated with reduced risk of CKD stages 4–5.

**Table 4:** Odds ratios from multinomial logistic regression models for CKD stage 3 and stages 4–5 per μm increase in thickness of retinal layers supplied by the retinal microvasculature

ETDRS segment	Right eye						Left eye					
	CKD stage 3 (vs. stage 1–2)			CKD stage 4–5 (vs. stage 1–2)			CKD stage 3 (vs. stage 1–2)			CKD stage 4–5 (vs. stage 1–2)		
	OR	95% CI	p	OR	95% CI	p	OR	95% CI	p	OR	95% CI	p
Nerve fibre layer												
N1	0.99	(0.86, 1.14)	0.88	0.88	(0.74, 1.05)	0.15	1.01	(0.90, 1.13)	0.93	1.00	(0.90, 1.11)	0.95
S1	1.05	(0.96, 1.16)	0.27	1.00	(0.90, 1.11)	0.97	1.03	(0.94, 1.13)	0.52	0.99	(0.89, 1.10)	0.85
T1	0.83	(0.64, 1.06)	0.14	1.04	(0.96, 1.13)	0.35	0.95	(0.77, 1.17)	0.63	1.14	(0.96, 1.36)	0.14
I1	0.97	(0.87, 1.08)	0.58	0.94	(0.84, 1.06)	0.31	1.02	(0.92, 1.13)	0.70	1.00	(0.90, 1.11)	0.94
Ganglion cell layer												
N1	1.02	(0.96, 1.09)	0.48	0.93	(0.87, 1.00)	0.04*	1.00	(0.94, 1.07)	0.98	0.94	(0.87, 1.01)	0.08
S1	1.02	(0.95, 1.09)	0.60	0.92	(0.86, 0.99)	0.02*	1.01	(0.94, 1.09)	0.75	0.95	(0.88, 1.03)	0.18
T1	1.01	(0.94, 1.08)	0.87	0.89	(0.82, 0.97)	0.01*	1.01	(0.95, 1.07)	0.74	0.93	(0.87, 0.99)	0.02*
I1	1.02	(0.95, 1.10)	0.57	0.90	(0.83, 0.96)	0.004*	1.03	(0.96, 1.10)	0.47	0.90	(0.84, 0.97)	0.004*
Inner plexiform layer												
N1	1.01	(0.92, 1.11)	0.83	0.91	(0.82, 1.00)	0.04*	0.99	(0.91, 1.09)	0.90	0.90	(0.82, 1.00)	0.04*
S1	1.01	(0.92, 1.12)	0.78	0.89	(0.80, 0.99)	0.03*	0.99	(0.89, 1.10)	0.81	0.88	(0.78, 0.99)	0.03*
T1	1.00	(0.90, 1.11)	0.96	0.87	(0.78, 0.98)	0.02*	1.03	(0.93, 1.13)	0.61	0.89	(0.80, 0.99)	0.03*
I1	1.01	(0.90, 1.12)	0.92	0.85	(0.76, 0.96)	0.01*	1.02	(0.92, 1.14)	0.66	0.82	(0.73, 0.93)	0.002*
Inner nuclear layer												
N1	0.91	(0.82, 1.02)	0.10	0.9	(0.79, 1.02)	0.11	0.90	(0.81, 1.01)	0.07	0.96	(0.86, 1.06)	0.42
S1	0.92	(0.83, 1.02)	0.11	0.9	(0.80, 1.01)	0.08	0.92	(0.84, 1.01)	0.09	0.88	(0.79, 0.97)	0.01*
T1	0.91	(0.80, 1.03)	0.14	0.87	(0.75, 1.00)	0.06	0.93	(0.83, 1.03)	0.17	0.88	(0.78, 0.99)	0.03*
I1	0.96	(0.86, 1.06)	0.42	0.96	(0.84, 1.08)	0.49	0.93	(0.84, 1.03)	0.16	0.89	(0.79, 1.00)	0.06

*ETDRS* Early Treatment Diabetic Retinopathy Study, *OR* Odds ratio, *SD* Standard deviation, *CI* Confidence interval, *F* Fovea, *S1* Superior segment 1, *N1* Nasal segment 1, *I1* Inferior segment 1, *T1* Temporal segment 1.*significant values. Adjustment for age, mean arterial blood pressure, diabetes status, low-density lipoprotein, body mass index, and sex

### Associations between retinal thickness and the choroidal vasculature

ORs for CKD were estimated per μm change for retinal layers supplied primarily by the choroidal vasculature (OPL, ONL, RPE) for annulus 1 ETDRS segments with adjustment for age, MABP, diabetes, LDL, BMI, and sex (Table 5). No significant associations were detected between CKD and any of the outer retinal layers in annulus 1 or 2 in adjusted analyses. Thickness of the foveal segment of the outer nuclear area was negatively associated with risk of CKD stages 3–4 following adjustment for age, MABP, diabetes, LDL, BMI, and sex.

**Table 5:** Odds ratios from multinomial logistic regression models for CKD stage 3 and stages 4–5 per μm increase in thickness of retinal layers supplied by the choroidal vasculature

ETDRS segment	Right eye						Left eye					
	CKD stage 3 (vs. stage 1–2)			CKD stage 4–5 (vs. stage 1–2)			CKD stage 3 (vs. stage 1–2)			CKD stage 4–5 (vs. stage 1–2)		
	OR	95% CI	p	OR	95% CI	p	OR	95% CI	p	OR	95% CI	p
Outer plexiform layer												
N1	1.03	(0.97, 1.10)	0.33	1.03	(0.96, 1.11)	0.45	1.00	(0.94, 1.07)	0.89	1.00	(0.93, 1.08)	0.94
S1	1.02	(0.96, 1.09)	0.46	0.95	(0.86, 1.04)	0.26	1.00	(0.95, 1.06)	0.93	0.93	(0.87, 0.99)	0.03*
T1	1.07	(0.96, 1.20)	0.22	0.93	(0.81, 1.08)	0.34	1.11	(1.02, 1.22)	0.02*	1.01	(0.90, 1.13)	0.84
I1	1.01	(0.94, 1.08)	0.78	1.05	(0.98, 1.14)	0.18	1.04	(0.97, 1.12)	0.24	1.08	(1.01, 1.16)	0.03*
Outer nuclear layer												
N1	0.98	(0.94, 1.01)	0.22	0.95	(0.91, 1.00)	0.05*	0.99	(0.96, 1.02)	0.35	0.97	(0.93, 1.01)	0.10
S1	0.99	(0.95, 1.03)	0.52	0.97	(0.92, 1.02)	0.23	0.99	(0.97, 1.02)	0.45	0.99	(0.96, 1.03)	0.61
T1	1.00	(0.96, 1.04)	0.89	1.00	(0.97, 1.04)	0.78	0.98	(0.94, 1.01)	0.20	0.98	(0.95, 1.02)	0.42
I1	0.99	(0.95, 1.03)	0.68	0.96	(0.91, 1.01)	0.10	0.96	(0.92, 1.00)	0.07	0.94	(0.89, 0.98)	0.01*
Retinal pigmented epithelium												
N1	1.02	(0.80, 1.30)	0.88	1.00	(0.75, 1.35)	0.98	1.21	(0.96, 1.53)	0.11	1.01	(0.76, 1.35)	0.93
S1	1.09	(0.84, 1.42)	0.51	0.97	(0.70, 1.35)	0.87	1.13	(0.91, 1.39)	0.26	0.77	(0.55, 1.07)	0.11
T1	1.04	(0.80, 1.35)	0.79	1.05	(0.76, 1.44)	0.78	1.09	(0.84, 1.41)	0.53	1.01	(0.74, 1.38)	0.96
I1	0.94	(0.74, 1.20)	0.63	1.00	(0.75, 1.34)	0.99	1.09	(0.91, 1.31)	0.34	0.99	(0.77, 1.29)	0.96

*ETDRS* Early Treatment Diabetic Retinopathy Study, *OR* Odds ratio, *SD* Standard deviation, *CI* Confidence interval, *F* Fovea, *S1* Superior segment 1, *N1* Nasal segment 1, *I1* Inferior segment 1, *T1* Temporal segment 1.*significant values. Adjustment for age, mean arterial blood pressure, diabetes status, low-density lipoprotein, body mass index, and sex

### Retinal microvascular and choroidal measures

ORs for CKD per SD change in choroidal and retinal microvascular parameters are presented (Table 6). No significant associations were detected between CVI and CKD. Higher choroidal volumes were significantly associated with lower risk of CKD stage 3 in unadjusted analyses but the association was no longer significant following adjustment for age, MABP, diabetes, LDL, BMI and sex. For each SD increase in choroidal volume in the unadjusted analysis, the odds ratio for CKD stage 3 were 0.55 times that for stage 1–2 (OR 0.55, 95% CI 0.36, 0.83).

**Table 6:** Odds ratios for CKD stage 3 and stages 4–5 per standard deviation increase choroidal vascularity index (unit-less), Choroidal volume (mm^3^), AVR (unit-less), and fractal dimension

Variable	CKD stage 3 (vs. stage 1–2)			CKD stage 4–5 (vs. stage 1–2)		
	OR	95% CI	p	OR	95% CI	p
Choroidal vascularity index	1.37	(0.80, 2.36)	0.25	1.44	(0.78, 2.67)	0.25
Choroidal Volume (mm^3^)	0.90	(0.55, 1.46)	0.67	0.76	(0.40, 1.44)	0.40
CRAE	0.92	(0.54, 1.55)	0.75	0.66	(0.35, 1.28)	0.22
CRVE	0.66	(0.41, 1.06)	0.08	1.07	(0.55, 2.07)	0.85
AVR	1.67	(1.01, 2.75)	0.05*	0.59	(0.27, 1.26)	0.17
LogFractalDimCa1	1.05	(0.62, 1.79)	0.86	0.45	(0.24, 0.84)	0.01*
LogFractalDimCv1	0.94	(0.58, 1.54)	0.82	0.53	(0.29, 0.94)	0.03*

*CI* Confidence interval, *OR* Odds ratio. *significant values. Adjustment for age, mean arterial blood pressure, diabetes status, low-density lipoprotein, body mass index, and sex

Retinal microvascular changes were associated with CKD. Greater venular diameter (higher CRVE) was associated with lower OR for CKD stage 3 in the unadjusted analysis. For each SD increase in CRVE in the unadjusted analysis, the odds ratio for CKD stage 4–5 were 0.67 times that for stage 1–2 (OR 0.67, 95% CI 0.46, 0.98). No significant associations were detected between retinal arteriolar calibre (CRAE) and CKD. However, AVR (the ratio between arteriolar and venular calibre) was positively associated with CKD stage 3 in both unadjusted and adjusted analyses and CKD stages 4–5 in the unadjusted model only. Arteriolar fractal dimension was negatively associated with risk of CKD stages 4–5 in both adjusted and unadjusted analyses, and CKD stage 3 in the unadjusted analysis only. Venular fractal dimension was not significantly associated with CKD stage 3, but was negatively associated with risk of CKD stages 4–5 in both adjusted and unadjusted analyses.

## Discussion

We assessed associations between the thickness of the retinal layers measured using SD-OCT, and CKD stages in a population with multiple comorbidities. Thinner retinas were associated with CKD stages 4–5. For example, per μm increase in thickness of the full retina in segment N1, the odds of CKD stage 4–5 were reduced by 3% per μm (i.e. OR of 0.97) compared with CKD stage 1–2. Differences in retinal thickness were primarily as a consequence of lower thickness of the inner retinal layers. In particular, thinner GCL and IPL were associated with CKD stages 4–5, with associations being largely limited to the proximal macular segments of annulus 1 as differentiated by the ETDRS grid. These associations were significant following adjustment for a range of important potential confounding factors such as age, blood pressure, diabetes status, LDL, BMI, and sex and support and expand previously reported findings [25]. Of note, significant associations were detected only within layers supported by the retinal microvascular blood supply, and not the layers supported by a choroidal blood supply. No significant associations were found bilaterally between retinal thickness and earlier stage CKD (CKD stage 3). This finding does not support the hypothesis that changes to retinal thickness may be detectible early in the progression of CKD. Collectively these data suggest that retinal thickness, and in particular thickness of the IPL and GCL, is lower in those with CKD stages 4–5 independent of diabetes, blood pressure and other potential confounding factors.

Similar changes have been reported in studies of diabetes and its complications. Thinning of the inner retinal layers, specifically the IPL, INL, and GCL, within annulus 1 has previously been associated with diabetes in those with early stages of diabetic retinopathy [33, 34]. Murine models of early diabetes have also shown thinning of the INL and IPL in association with reductions in retinal ganglion cell numbers coinciding with retinal neuronal and vascular apoptosis [35], which may reflect the susceptibility of retinal vascular and neuronal tissues to similar noxious environments. Similar patterns of retinal apoptosis have also been observed in human retinas post mortem from individuals with diabetes, including those without retinal pathology [35].

Potential confounding factors such as age [36–40], LDL [40], MABP [29], sex [36, 37, 40–42], and BMI [41] have all been previously associated with retinal thickness and as such were considered as potential confounders. These factors are also associated with vascular risk, and indeed vascular damage has been proposed as a mechanism that contributes to changes in retinal thickness in individuals with diabetes. However, the cellular, biochemical and physiological mechanisms that lead to neural cell loss and vascular changes within the diabetic retina have still to be fully determined [43, 44]. Given the cross-sectional nature of this study, the temporality of events relating to cause and effect in those with CKD stages 4–5 cannot be established and longitudinal data would be necessary to determine causality. However, this study provides novel evidence of these associations with individual retinal layers, and thus some insight to the mechanisms behind previously reported changes in retinal thickness in those with CKD [25].

Several studies have reported structural alterations in the retinal vasculature in association with CKD and reduced renal function [18, 19, 23, 45, 46]. Furthermore, inflammation and hypoxia have been linked to impaired metabolism in Müller cells which help protect against ganglion cell apoptosis [47], supporting the hypothesis that retinal neurodegeneration in those with reduced renal function may result from vascular impairment. Vascular impairment may also explain the physical pattern of associations observed. The outer retinal layers receive nutrients via diffusion from the choroid, while the inner layers are serviced by the retinal microvasculature. Consequently, the inner retinal layers are more susceptible to hypoxic injury compared to the outer layers [48]. Hypoxic damage has also been linked to retinal ganglion cell death mediated by inflammatory cytokines [48], consistent with the findings of lower GCL thickness in those with CKD stages 4–5. Indeed, all of the significant associations detected bilaterally were detected in layers primarily supplied by the retinal microvasculature and not the choroid.

The proximal annulus 1, comprising segments S1, N1, I1, and T1, is in an area approaching the foveal avascular zone. Segments of the retina proximal to, but not within, the foveal segment, thus have a sparser retinal vasculature and are more likely to be affected. The absence of associations between foveal thickness and renal status may be as a consequence of its choroidal blood supply [49] protecting this tissue from susceptibility to hypoxia from retinal vascular impairment. Associations will also be affected by the smaller size and partial coverage of the inner retinal layers over this area of the retina [50].

Retinal neurodegeneration in diabetes has been associated with NFL thinning related to capillary occlusion and retinal ganglion cell loss [51], and thinner NFLs have been reported in patients with CKD undergoing haemodialysis or peritoneal dialysis [52]. It is therefore unclear why we did not observe lower NFL thickness in CKD stages 4–5, although a lack of significant variation in NFL thickness between CKD cases and healthy controls has previously been reported in earlier stages of CKD [25]. Interestingly, in regression analyses using eGFR as a continuous outcome variable, we found a significantly thicker NFL bilaterally in the distal temporal segment in association with higher eGFR (Additional file 1: Table S2). In optic disc oedema, the NFL thickens at an early stage of the pathology but thins as the disease progresses [53]. Given a lack of association between NFL and CKD has been reported in a relatively healthy CKD sample previously [25], the negative association observed between eGFR and NFL thickness in this study population with a higher comorbidity burden may represent the early stages of neurodegeneration prior to significant axonal loss, and axonal loss may increase as the pathology develops. NFL thickening, without concomitant thickening of the other retinal layers (as in our study), has been reported in inflammatory optic neuropathies [54] and is not without precedent, although the potential for a type-1 error must be considered. The temporal segments comprise an area of the retina which typically has less coverage of arterioles and venules, being distal from the optic disc, and further from the main branches of the retinal arcades, which may increase its susceptibility to vascular damage.

We also assessed choroidal volume and CVI and detected an association between choroidal volume and CKD stage 3 in an unadjusted analysis, although this was no longer significant following adjustment for potential confounders. The association between choroidal volume may be explained by well-known CVD risk factors (age, sex, BMI, MABP, LDL, and diabetes) reflecting the vascular nature of this tissue. This finding is consistent with the possible role of the retinal microvasculature (as opposed to the choroidal circulation) in the inner retinal changes observed and the lack of outer retinal changes. Moreover, retinal microvascular branching patterns (measured as fractal dimension) were significantly associated with CKD stage. Those with more extensive microvascular branching had lower risk of CKD stages 4–5.

This study had several strengths including the ability to control for major confounders such as age, sex, BMI, diabetes, LDL and MABP. Given that many of the associations remained significant following adjustment, it may be that the observed associations, and previously reported unadjusted associations [25], between retinal thickness and renal function manifest as a consequence of a CKD specific mechanistic pathway.

The use of SD-OCT and the HEYEX semi-automated software provided highly reliable measures that enabled a more sensitive evaluation of the retinal and choroidal layers than previously reported. Furthermore, the subsequent correction by graders blinded to participant characteristics reduced the influence of observer bias in this study. To the best of our knowledge, this was the first study to investigate the direct association between such a broad range of retinal and choroidal layers and eGFR. SD-OCT provided robust differentiation of the retinal layers that will help improve our understanding of the cellular pathways behind the associations observed. SD-OCT-EDI enabled evaluation of choroidal measures including choroidal volume and CVI to improve the sensitivity of the choroidal measures [25] that are frequently affected by unevenness of the choroidal-scleral interface. We have combined the choroidal measures with retinal microvascular assessment to provide insight into potential vascular mediation of retinal thickness associated with CKD. Moreover, OCT is currently used in the diagnosis and evaluation of a variety of retinal conditions, such as diabetic retinopathy, and thus retinal changes associated with CKD may be assessed using widely available technology.

There were several limitations in this study. The cross-sectional nature of this study does not allow for the determination of causality of association. eGFR lacks sensitivity as an indicator of renal decline [55], and so the clinical relevance of these findings require further consideration through longitudinal evaluation of changes in retinal thickness with declining kidney function with age. Furthermore, the specificity of the observed associations with the underlying cause of CKD was not examined. Future studies may investigate the specificity of changes in retinal thickness with disorders such as diabetic nephropathy to determine the value of such retinal changes in risk stratification. This might inform the use of more invasive procedures, such as renal biopsy.

Additionally, assessment of proteinuria, underlying CKD cause, measures of systemic vascular health and use of diuretics may inform potential mechanisms and improve predictive capacity. In particular, future work should consider measures of proteinuria and diuretic use to determine potential influence on retinal thickness. Furthermore, because the aetiology of renal decline was not assessed in the present study, it is unclear whether patterns of retinal thinning in CKD depend on the underlying cause of reduced renal function. The effect of the associations considered are not generalisable to the general population as recruitment was undertaken in a clinical setting. CKD is defined clinically as persistently decreased eGFR of less than 60 mL/min/1.73 m^2^ for at least 3 months, and / or persistent proteinuria [55]. This differs from the definition we used in this study which relied solely on a single measure of renal function and associations may therefore be weaker than those in a study using stricter clinical CKD staging.

Retinal thickness is related to a variety of other conditions. For example, reduced thickness of the retinal ganglion cell complex (GCC; a composite retinal layer comprising the retinal layers containing the ganglion cell dendritic synapses with bipolar and amacrine cells, the ganglion cell bodies, and the ganglion cell axons viz., the IPL, GCL, and NFL respectively) has also been associated with a variety of ocular diseases in their early stages, such as glaucoma [56], retinal vessel loss in open-angle glaucoma [57], and chiasmal compression [58]. Similar changes have also been reported in early age-related macular degeneration [59]. This highlights the sensitivity of these layers to a variety of pathologies and also indicates the need to control for these conditions, where possible, in future analyses. Choroidal thickness is also affected by other factors such as diurnal variation and is affected by fluid intake [60]. We did not record or control for fluid intake and this may represent an unaccounted confounding influence. However, all measurements were made in the afternoon, and so diurnal variation is thought to have had little effect on the associations observed. Finally, the issue of multiple testing was a limitation. The number of statistical associations evaluated provides an increased risk of type-1 error. However, we have limited our conclusions and discussion to reflect associations with bilateral significance only in an effort to mitigate such influences.

## Conclusion

Reduced retinal thickness, and in particular a thinner inner retinal layer, was found to be associated with CKD stage 4–5 independent of other important risk factors (age, MABP, diabetes status, LDL, BMI and sex). These associations were limited to layers of the retina supplied by the retinal microvasculature and to areas immediately surrounding the foveal zone. These findings do not support the hypothesis that changes in retinal thickness are detectible at the earliest stages of CKD, but highlight a distinct pattern of retinal changes detectable in CKD stages 4–5. In particular, IPL and GCL thickness are lower in those with CKD stages 4–5 and occur alongside changes to retinal microvascular AVR and fractal dimension.

## Supplementary information

**Additional file 1:** **Table S1.** Odds ratios from multinomial logistic regression models for CKD stage 3 and stages 4–5 per μm increase in thickness of retinal layers supplied by the retinal microvasculature. **Table S2.** Regression coefficients (β) between thickness (μm) of the retinal layers with eGFR (ml/min/1.73m^2^) for ETDRS grid annulus 1.

## Data Availability

The datasets generated and/or analysed during the current study are not publicly available due the ethical requirement to protect participant anonymity but are available from the corresponding author on reasonable request.

## Abbreviations

ART: Automatic real-time tracking
AVR: Arteriovenous ratio
BMI: Body mass index
CI: 95% confidence intervals
CKD: Chronic kidney disease
CRAE: Central retinal arteriolar equivalent
CRVE: Central retinal venular equivalent
CVD: Cardiovascular disease
CVI: Choroidal vascularity index
EDI: SD-OCT enhanced depth imaging
eGFR: Estimated glomerular filtration rate
ETDRS: Early Treatment Diabetic Retinopathy Study
F: Foveal segment
GCL: Ganglion cell layer
HNFL-ONL: Henle’s nerve fibre layer and outer nuclear layer
I: Inferior segment
IN*OCT: International Nomenclature for Optical Coherence Tomography
INL: Inner nuclear layer
IPL: Inner plexiform layer
IRL: Inner retinal layer
LDL: Diabetes status, low-density lipoprotein
MABP: Mean arterial blood pressure
N: Nasal segment
NFL: Nerve fibre layer
OCT: Optical coherence tomography
OPL: Outer plexiform layer
OR: Odds ratios
ORL: Outer retinal layer
RPE: Retinal pigment epithelium
S: Superior segment
SD: Standard deviation
SD-OCT: Spectral domain optical coherence tomography
T: Temporal segment
