# Supplementary material for: Association of reduced inner retinal thicknesses with chronic kidney disease
Source: BMC Nephrol. 2020 Jan 31;21:37. doi: 10.1186/s12882-019-1679-1 (PMC6995224; doi:10.1186/s12882-019-1679-1)
Supplement: Supplementary file 1 — Additional file 1: Table S1. Odds ratios from multinomial logistic regression models for CKD stage 3 and stages 4–5 per μm increase in thickness of retinal layers supplied by the retinal microvasculature. Table S2. Regression coefficients (β) between thickness (μm) of the retinal layers with eGFR (ml/min/1.73m2) for ETDRS grid annulus 1. [file 12882_2019_1679_MOESM1_ESM.docx]

**Supplementary material**

**Title:** Association of reduced inner retinal thicknesses with Chronic Kidney Disease

Running title: Retinal thickness and renal function.

Authors: Euan N Paterson BSc,^1^ Meera L Ravindran BSc,^1^ Kayleigh Griffiths PhD,^1^ Claire A Le Velley BSc,^1^ Chris C Cardwell PhD,^1^ Rachel McCarter MSc,^1^ Patrick Nicol BSc,^1^ Jay K Chhablani MD,^2^ Thomas J MacGillivray PhD,^3^ Mark Harbinson MD,^4^ Alexander P Maxwell MD,^1^ Ruth E Hogg PhD,^1^ Gareth J McKay PhD^1^*

**Table S1:** Odds ratios from multinomial logistic regression models for CKD stage 3 and stages 4-5 per µm increase in thickness of retinal layers supplied by the retinal microvasculature.

|  | **Unadjusted** | | | | | | |  | **Adjusted** | | | | | | |
| --- | --- | --- | --- | --- | --- | --- | --- | --- | --- | --- | --- | --- | --- | --- | --- |
|  | CKD stage 3 (vs. stage 1-2) | | |  | CKD stage 4-5 (vs. stage 1-2) | | |  | CKD stage 3 (vs. stage 1-2) | | |  | CKD stage 4-5 (vs. stage 1-2) | | |
| ETDRS segment | OR | 95% CI | p |  | OR | 95% CI | p |  | OR | 95% CI | p |  | OR | 95% CI | p |
| **Right eye** |  |  |  |  |  |  |  |  |  |  |  |  |  |  |  |
| Retinal pigmented epithelium | | | | | | | | | | | | | | | |
| F (aka C0) | 0.93 | (0.79 , 1.11) | 0.44 |  | 1.06 | (0.91 , 1.23) | 0.45 |  | 1.04 | (0.87 , 1.24) | 0.69 |  | 1.12 | (0.93 , 1.36) | 0.24 |
| N1 | 0.87 | (0.72 , 1.07) | 0.19 |  | 0.88 | (0.69 , 1.13) | 0.31 |  | 1.02 | (0.80 , 1.30) | 0.88 |  | 1.00 | (0.75 , 1.35) | 0.98 |
| N2 | 0.81 | (0.64 , 1.02) | 0.07 |  | 0.83 | (0.63 , 1.10) | 0.20 |  | 0.85 | (0.64 , 1.14) | 0.29 |  | 0.87 | (0.60 , 1.26) | 0.46 |
| S1 | 0.94 | (0.76 , 1.16) | 0.57 |  | 0.87 | (0.67 , 1.12) | 0.28 |  | 1.09 | (0.84 , 1.42) | 0.51 |  | 0.97 | (0.70 , 1.35) | 0.87 |
| S2 | 0.99 | (0.78 , 1.26) | 0.95 |  | 0.81 | (0.60 , 1.10) | 0.18 |  | 1.01 | (0.76 , 1.36) | 0.92 |  | 0.83 | (0.58 , 1.20) | 0.33 |
| T1 | 0.91 | (0.73 , 1.13) | 0.38 |  | 0.88 | (0.67 , 1.16) | 0.38 |  | 1.04 | (0.80 , 1.35) | 0.79 |  | 1.05 | (0.76 , 1.44) | 0.78 |
| T2 | 1.06 | (0.95 , 1.18) | 0.28 |  | 0.76 | (0.53 , 1.07) | 0.11 |  | 1.02 | (0.88 , 1.19) | 0.79 |  | 0.82 | (0.55 , 1.23) | 0.34 |
| I1 | 0.88 | (0.71 , 1.07) | 0.20 |  | 0.91 | (0.71 , 1.17) | 0.48 |  | 0.94 | (0.74 , 1.20) | 0.63 |  | 1.00 | (0.75 , 1.34) | 0.99 |
| I2 | 0.91 | (0.71 , 1.17) | 0.47 |  | 0.81 | (0.59 , 1.12) | 0.20 |  | 0.92 | (0.67 , 1.25) | 0.58 |  | 0.83 | (0.57 , 1.23) | 0.36 |
| Outer retinal layer |  |  |  |  |  |  |  |  |  |  |  |  |  |  |  |
| F (aka C0) | 1.00 | (0.95 , 1.06) | 0.92 |  | 0.91 | (0.83 , 1.00) | 0.05 |  | 1.05 | (0.96 , 1.15) | 0.28 |  | 0.97 | (0.86 , 1.10) | 0.67 |
| N1 | 1.02 | (0.92 , 1.12) | 0.72 |  | 0.93 | (0.81 , 1.06) | 0.27 |  | 1.08 | (0.96 , 1.22) | 0.22 |  | 1.01 | (0.86 , 1.18) | 0.93 |
| N2 | 0.99 | (0.88 , 1.12) | 0.88 |  | 0.92 | (0.78 , 1.08) | 0.30 |  | 1.00 | (0.85 , 1.17) | 0.99 |  | 0.95 | (0.78 , 1.17) | 0.64 |
| S1 | 1.04 | (0.93 , 1.16) | 0.53 |  | 0.93 | (0.80 , 1.07) | 0.32 |  | 1.07 | (0.93 , 1.22) | 0.34 |  | 1.00 | (0.84 , 1.18) | 0.96 |
| S2 | 1.04 | (0.92 , 1.18) | 0.54 |  | 0.83 | (0.71 , 0.98) | 0.03* |  | 1.00 | (0.85 , 1.16) | 0.96 |  | 0.85 | (0.69 , 1.03) | 0.10 |
| T1 | 1.04 | (0.93 , 1.15) | 0.51 |  | 0.91 | (0.78 , 1.05) | 0.19 |  | 1.10 | (0.96 , 1.25) | 0.19 |  | 1.01 | (0.84 , 1.20) | 0.94 |
| T2 | 1.09 | (0.99 , 1.20) | 0.08 |  | 0.88 | (0.73 , 1.05) | 0.14 |  | 1.09 | (0.93 , 1.29) | 0.27 |  | 0.93 | (0.75 , 1.15) | 0.50 |
| I1 | 1.00 | (0.90 , 1.12) | 0.95 |  | 0.97 | (0.85 , 1.12) | 0.68 |  | 1.05 | (0.92 , 1.21) | 0.46 |  | 1.05 | (0.89 , 1.25) | 0.54 |
| I2 | 1.02 | (0.91 , 1.15) | 0.69 |  | 0.82 | (0.69 , 0.98) | 0.03 |  | 1.02 | (0.87 , 1.19) | 0.85 |  | 0.85 | (0.69 , 1.05) | 0.14 |
| Inner plexiform layer |  |  |  |  |  |  |  |  |  |  |  |  |  |  |  |
| F (aka C0) | 0.97 | (0.88 , 1.06) | 0.46 |  | 0.81 | (0.71 , 0.93) | 0.002* |  | 0.97 | (0.87 , 1.09) | 0.60 |  | 0.82 | (0.7 , 0.96) | 0.02* |
| N1 | 0.96 | (0.90 , 1.03) | 0.28 |  | 0.89 | (0.83 , 0.96) | 0.002* |  | 1.01 | (0.92 , 1.11) | 0.83 |  | 0.91 | (0.82 , 10) | 0.04 |
| N2 | 1.02 | (0.92 , 1.13) | 0.77 |  | 0.94 | (0.83 , 1.07) | 0.38 |  | 1.08 | (0.95 , 1.23) | 0.23 |  | 0.93 | (0.8 , 1.07) | 0.31 |
| S1 | 0.98 | (0.91 , 1.06) | 0.61 |  | 0.89 | (0.82 , 0.96) | 0.004* |  | 1.01 | (0.92 , 1.12) | 0.78 |  | 0.89 | (0.8 , 0.99) | 0.03* |
| S2 | 1.06 | (0.94 , 1.21) | 0.33 |  | 0.90 | (0.77 , 1.04) | 0.16 |  | 1.10 | (0.94 , 1.29) | 0.23 |  | 0.91 | (0.76 , 1.08) | 0.26 |
| T1 | 0.97 | (0.90 , 1.05) | 0.43 |  | 0.88 | (0.81 , 0.95) | 0.002* |  | 1.00 | (0.9 , 1.11) | 0.96 |  | 0.87 | (0.78 , 0.98) | 0.02* |
| T2 | 0.97 | (0.88 , 1.08) | 0.63 |  | 0.94 | (0.83 , 1.07) | 0.39 |  | 1.01 | (0.89 , 1.15) | 0.82 |  | 0.98 | (0.86 , 1.13) | 0.83 |
| I1 | 0.95 | (0.88 , 1.03) | 0.23 |  | 0.84 | (0.77 , 0.92) | <0.001* |  | 1.01 | (0.9 , 1.12) | 0.92 |  | 0.85 | (0.76 , 0.96) | 0.01* |
| I2 | 0.99 | (0.88 , 1.11) | 0.83 |  | 0.95 | (0.82 , 1.10) | 0.47 |  | 1.06 | (0.92 , 1.21) | 0.45 |  | 0.96 | (0.82 , 1.13) | 0.65 |
| Full retinal thickness |  |  |  |  |  |  |  |  |  |  |  |  |  |  |  |
| F (aka C0) | 1.00 | (0.98 , 1.01) | 0.57 |  | 0.97 | (0.95 , 0.99) | 0.002* |  | 0.99 | (0.97 , 1.01) | 0.53 |  | 0.97 | (0.94 , 0.99) | 0.01* |
| N1 | 0.99 | (0.97 , 1.01) | 0.34 |  | 0.96 | (0.94 , 0.98) | <0.001* |  | 1.00 | (0.97 , 1.02) | 0.89 |  | 0.97 | (0.94 , 0.99) | 0.02* |
| N2 | 1.00 | (0.98 , 1.02) | 0.73 |  | 0.97 | (0.95 , 0.99) | 0.02* |  | 1.01 | (0.98 , 1.03) | 0.50 |  | 0.98 | (0.95 , 1.00) | 0.11 |
| S1 | 1.00 | (0.98 , 1.01) | 0.63 |  | 0.96 | (0.94 , 0.98) | <0.001* |  | 1.00 | (0.98 , 1.03) | 0.76 |  | 0.97 | (0.94 , 0.99) | 0.01* |
| S2 | 1.01 | (0.99 , 1.03) | 0.4 |  | 0.97 | (0.95 , 1.00) | 0.05* |  | 1.02 | (0.99 , 1.04) | 0.25 |  | 0.98 | (0.95 , 1.01) | 0.20 |
| T1 | 0.99 | (0.98 , 1.01) | 0.56 |  | 0.98 | (0.96 , 1.00) | 0.03* |  | 1.00 | (0.98 , 1.02) | 0.80 |  | 0.99 | (0.97 , 1.01) | 0.33 |
| T2 | 1.00 | (0.99 , 1.02) | 0.74 |  | 1.00 | (0.99 , 1.02) | 0.61 |  | 1.01 | (0.99 , 1.03) | 0.43 |  | 1.01 | (0.99 , 1.02) | 0.54 |
| I1 | 0.99 | (0.97 , 1.01) | 0.26 |  | 0.96 | (0.94 , 0.98) | 0* |  | 1.00 | (0.97 , 1.02) | 0.87 |  | 0.97 | (0.95 , 1.00) | 0.02* |
| I2 | 1.00 | (0.98 , 1.02) | 0.87 |  | 0.99 | (0.96 , 1.01) | 0.27 |  | 1.00 | (0.98 , 1.03) | 0.72 |  | 0.99 | (0.97 , 1.02) | 0.56 |
| Inner retinal layer |  |  |  |  |  |  |  |  |  |  |  |  |  |  |  |
| F (aka C0) | 1.00 | (0.98 , 1.01) | 0.60 |  | 0.97 | (0.95 , 0.99) | 0.01* |  | 0.99 | (0.97 , 1.01) | 0.34 |  | 0.96 | (0.94 , 0.99) | 0.01* |
| N1 | 0.99 | (0.97 , 1.01) | 0.31 |  | 0.96 | (0.94 , 0.98) | <0.001* |  | 1.00 | (0.97 , 1.02) | 0.70 |  | 0.97 | (0.94 , 0.99) | 0.01* |
| N2 | 1.00 | (0.98 , 1.02) | 0.77 |  | 0.97 | (0.95 , 1.00) | 0.02* |  | 1.01 | (0.98 , 1.03) | 0.54 |  | 0.98 | (0.95 , 1.00) | 0.11 |
| S1 | 0.99 | (0.97 , 1.01) | 0.56 |  | 0.96 | (0.94 , 0.98) | <0.001* |  | 1.00 | (0.98 , 1.03) | 0.87 |  | 0.97 | (0.94 , 0.99) | 0.01* |
| S2 | 1.01 | (0.99 , 1.03) | 0.46 |  | 0.98 | (0.95 , 1.00) | 0.10 |  | 1.02 | (0.99 , 1.05) | 0.24 |  | 0.98 | (0.95 , 1.01) | 0.28 |
| T1 | 0.99 | (0.98 , 1.01) | 0.50 |  | 0.98 | (0.96 , 1.00) | 0.05* |  | 1.00 | (0.98 , 1.02) | 0.92 |  | 0.99 | (0.96 , 1.01) | 0.34 |
| T2 | 1.00 | (0.98 , 1.02) | 0.98 |  | 1.01 | (0.99 , 1.02) | 0.50* |  | 1.01 | (0.99 , 1.03) | 0.53 |  | 1.01 | (0.99 , 1.02) | 0.48 |
| I1 | 0.99 | (0.97 , 1.01) | 0.26 |  | 0.96 | (0.94 , 0.98) | <0.001* |  | 1.00 | (0.97 , 1.02) | 0.80 |  | 0.97 | (0.94 , 0.99) | 0.02 |
| I2 | 1.00 | (0.98 , 1.02) | 0.84 |  | 0.99 | (0.96 , 1.02) | 0.47 |  | 1.00 | (0.98 , 1.03) | 0.72 |  | 0.99 | (0.97 , 1.02) | 0.71 |
| Nerve fibre layer |  |  |  |  |  |  |  |  |  |  |  |  |  |  |  |
| F (aka C0) | 0.92 | (0.79 , 1.06) | 0.25 |  | 0.74 | (0.62 , 0.90) | 0.002* |  | 0.92 | (0.78 , 1.10) | 0.37 |  | 0.79 | (0.63 , 0.99) | 0.04* |
| N1 | 1.05 | (0.93 , 1.19) | 0.44 |  | 0.84 | (0.72 , 0.99) | 0.03* |  | 0.99 | (0.86 , 1.14) | 0.88 |  | 0.88 | (0.74 , 1.05) | 0.15 |
| N2 | 1.02 | (0.98 , 1.07) | 0.29 |  | 0.96 | (0.91 , 1.01) | 0.13 |  | 1.01 | (0.97 , 1.06) | 0.57 |  | 0.98 | (0.93 , 1.04) | 0.49 |
| S1 | 1.06 | (0.98 , 1.14) | 0.16 |  | 0.99 | (0.89 , 1.10) | 0.86 |  | 1.05 | (0.96 , 1.16) | 0.27 |  | 1.00 | (0.90 , 1.11) | 0.97 |
| S2 | 1.03 | (0.98 , 1.09) | 0.24 |  | 1.00 | (0.94 , 1.07) | 0.97 |  | 1.03 | (0.97 , 1.10) | 0.34 |  | 1.00 | (0.94 , 1.08) | 0.92 |
| T1 | 1.01 | (0.86 , 1.18) | 0.89 |  | 1.09 | (0.96 , 1.24) | 0.20 |  | 0.83 | (0.64 , 1.06) | 0.14 |  | 1.04 | (0.96 , 1.13) | 0.35 |
| T2 | 1.13 | (0.96 , 1.32) | 0.13 |  | 1.17 | (1.00 , 1.38) | 0.05 |  | 1.03 | (0.89 , 1.20) | 0.68 |  | 1.06 | (0.95 , 1.19) | 0.31 |
| I1 | 0.97 | (0.89 , 1.07) | 0.58 |  | 0.91 | (0.81 , 1.02) | 0.10 |  | 0.97 | (0.87 , 1.08) | 0.58 |  | 0.94 | (0.84 , 1.06) | 0.31 |
| I2 | 1.00 | (0.96 , 1.05) | 0.89 |  | 0.97 | (0.91 , 1.02) | 0.26 |  | 0.99 | (0.94 , 1.04) | 0.71 |  | 0.96 | (0.91 , 1.03) | 0.24 |
| Outer plexiform layer | | | | | | | | | | | | | | | |
| F (aka C0) | 1.02 | (0.96 , 1.08) | 0.52 |  | 0.96 | (0.88 , 1.04) | 0.31 |  | 1.02 | (0.95 , 1.10) | 0.55 |  | 0.98 | (0.89 , 1.07) | 0.59 |
| N1 | 1.03 | (0.98 , 1.08) | 0.25 |  | 1.02 | (0.96 , 1.09) | 0.47 |  | 1.03 | (0.97 , 1.10) | 0.33 |  | 1.03 | (0.96 , 1.11) | 0.45 |
| N2 | 1.08 | (0.96 , 1.21) | 0.21 |  | 0.99 | (0.84 , 1.16) | 0.89 |  | 1.13 | (0.96 , 1.32) | 0.14 |  | 1.00 | (0.82 , 1.20) | 0.97 |
| S1 | 1.00 | (0.95 , 1.05) | 0.88 |  | 0.91 | (0.83 , 0.99) | 0.04* |  | 1.02 | (0.96 , 1.09) | 0.46 |  | 0.95 | (0.86 , 1.04) | 0.26 |
| S2 | 1.04 | (0.92 , 1.17) | 0.51 |  | 0.87 | (0.72 , 1.04) | 0.13 |  | 1.12 | (0.96 , 1.30) | 0.14 |  | 0.94 | (0.77 , 1.14) | 0.53 |
| T1 | 1.01 | (0.93 , 1.11) | 0.76 |  | 0.85 | (0.74 , 0.99) | 0.04* |  | 1.07 | (0.96 , 1.20) | 0.22 |  | 0.93 | (0.81 , 1.08) | 0.34 |
| T2 | 0.98 | (0.82 , 1.16) | 0.78 |  | 0.90 | (0.72 , 1.13) | 0.38 |  | 1.08 | (0.87 , 1.35) | 0.47 |  | 1.05 | (0.83 , 1.34) | 0.67 |
| I1 | 1.02 | (0.96 , 1.08) | 0.56 |  | 1.03 | (0.96 , 1.11) | 0.38 |  | 1.01 | (0.94 , 1.08) | 0.78 |  | 1.05 | (0.98 , 1.14) | 0.18 |
| I2 | 1.09 | (0.95 , 1.26) | 0.21 |  | 1.07 | (0.90 , 1.27) | 0.44 |  | 1.12 | (0.93 , 1.34) | 0.23 |  | 1.09 | (0.90 , 1.33) | 0.37 |
| Inner nuclear layer |  |  |  |  |  |  |  |  |  |  |  |  |  |  |  |
| F (aka C0) | 1.00 | (0.94 , 1.05) | 0.93 |  | 1.00 | (0.93 , 1.07) | 0.99 |  | 0.95 | (0.88 , 1.02) | 0.16 |  | 0.98 | (0.90 , 1.08) | 0.72 |
| N1 | 0.92 | (0.84 , 1.00) | 0.05* |  | 0.88 | (0.79 , 0.98) | 0.02* |  | 0.91 | (0.82 , 1.02) | 0.10 |  | 0.90 | (0.79 , 1.02) | 0.11 |
| N2 | 0.99 | (0.88 , 1.12) | 0.87 |  | 0.99 | (0.86 , 1.15) | 0.94 |  | 1.04 | (0.91 , 1.19) | 0.58 |  | 1.03 | (0.87 , 1.21) | 0.72 |
| S1 | 0.91 | (0.84 , 0.99) | 0.04* |  | 0.87 | (0.78 , 0.97) | 0.02* |  | 0.92 | (0.83 , 1.02) | 0.11 |  | 0.90 | (0.80 , 1.01) | 0.08 |
| S2 | 1.01 | (0.90 , 1.14) | 0.82 |  | 0.96 | (0.82 , 1.12) | 0.60 |  | 1.09 | (0.93 , 1.28) | 0.29 |  | 1.00 | (0.83 , 1.20) | 1.00 |
| T1 | 0.92 | (0.83 , 1.01) | 0.08 |  | 0.88 | (0.78 , 0.99) | 0.03* |  | 0.91 | (0.80 , 1.03) | 0.14 |  | 0.87 | (0.75 , 1.00) | 0.06 |
| T2 | 0.96 | (0.85 , 1.08) | 0.49 |  | 1.00 | (0.87 , 1.14) | 1.00 |  | 1.05 | (0.90 , 1.22) | 0.56 |  | 1.07 | (0.92 , 1.24) | 0.36 |
| I1 | 0.94 | (0.86 , 1.03) | 0.17 |  | 0.93 | (0.84 , 1.04) | 0.22 |  | 0.96 | (0.86 , 1.06) | 0.42 |  | 0.96 | (0.84 , 1.08) | 0.49 |
| I2 | 0.97 | (0.85 , 1.11) | 0.64 |  | 1.13 | (0.99 , 1.29) | 0.06 |  | 1.02 | (0.86 , 1.21) | 0.84 |  | 1.16 | (0.97 , 1.40) | 0.10 |
| Outer nuclear layer |  |  |  |  |  |  |  |  |  |  |  |  |  |  |  |
| F (aka C0) | 0.99 | (0.96 , 1.02) | 0.55 |  | 0.94 | (0.91 , 0.98) | 0.001* |  | 0.98 | (0.94 , 1.02) | 0.35 |  | 0.94 | (0.89 , 0.98) | 0.01* |
| N1 | 0.98 | (0.95 , 1.01) | 0.30 |  | 0.96 | (0.92 , 0.99) | 0.02* |  | 0.98 | (0.94 , 1.01) | 0.22 |  | 0.95 | (0.91 , 1.00) | 0.05* |
| N2 | 0.97 | (0.93 , 1.02) | 0.22 |  | 0.94 | (0.89 , 1.00) | 0.04* |  | 0.97 | (0.92 , 1.02) | 0.25 |  | 0.94 | (0.88 , 1.00) | 0.05 |
| S1 | 0.99 | (0.96 , 1.03) | 0.68 |  | 0.98 | (0.94 , 1.02) | 0.25 |  | 0.99 | (0.95 , 1.03) | 0.52 |  | 0.97 | (0.92 , 1.02) | 0.23 |
| S2 | 0.99 | (0.94 , 1.04) | 0.62 |  | 0.96 | (0.90 , 1.02) | 0.17 |  | 0.99 | (0.93 , 1.05) | 0.78 |  | 0.95 | (0.89 , 1.03) | 0.22 |
| T1 | 0.99 | (0.96 , 1.03) | 0.72 |  | 1.01 | (0.97 , 1.04) | 0.77 |  | 1.00 | (0.96 , 1.04) | 0.89 |  | 1.00 | (0.97 , 1.04) | 0.78 |
| T2 | 1.00 | (0.97 , 1.04) | 0.95 |  | 1.01 | (0.99 , 1.04) | 0.29 |  | 1.01 | (0.96 , 1.05) | 0.80 |  | 1.01 | (0.98 , 1.04) | 0.45 |
| I1 | 0.99 | (0.95 , 1.02) | 0.43 |  | 0.96 | (0.92 , 1.00) | 0.04* |  | 0.99 | (0.95 , 1.03) | 0.68 |  | 0.96 | (0.91 , 1.01) | 0.10 |
| I2 | 0.98 | (0.93 , 1.03) | 0.46 |  | 0.98 | (0.92 , 1.05) | 0.58 |  | 1.00 | (0.94 , 1.06) | 0.95 |  | 1.00 | (0.94 , 1.06) | 0.97 |
| Ganglion cell layer |  |  |  |  |  |  |  |  |  |  |  |  |  |  |  |
| F (aka C0) | 0.98 | (0.91 , 1.05) | 0.53 |  | 0.95 | (0.86 , 1.05) | 0.35 |  | 0.98 | (0.90 , 1.07) | 0.71 |  | 0.98 | (0.90 , 1.07) | 0.60 |
| N1 | 0.99 | (0.94 , 1.04) | 0.73 |  | 0.93 | (0.88 , 0.98) | 0.004* |  | 1.02 | (0.96 , 1.09) | 0.48 |  | 0.93 | (0.87 , 1.00) | 0.04* |
| N2 | 1.01 | (0.93 , 1.09) | 0.83 |  | 0.92 | (0.84 , 1.01) | 0.09 |  | 1.06 | (0.96 , 1.17) | 0.28 |  | 0.88 | (0.78 , 0.99) | 0.04* |
| S1 | 1.00 | (0.95 , 1.05) | 0.97 |  | 0.93 | (0.88 , 0.98) | 0.01* |  | 1.02 | (0.95 , 1.09) | 0.60 |  | 0.92 | (0.86 , 0.99) | 0.02* |
| S2 | 1.03 | (0.93 , 1.14) | 0.56 |  | 0.91 | (0.81 , 1.01) | 0.09 |  | 1.07 | (0.94 , 1.21) | 0.33 |  | 0.90 | (0.78 , 1.04) | 0.14 |
| T1 | 0.99 | (0.94 , 1.04) | 0.69 |  | 0.91 | (0.86 , 0.96) | <0.001* |  | 1.01 | (0.94 , 1.08) | 0.87 |  | 0.89 | (0.82 , 0.97) | 0.01* |
| T2 | 0.99 | (0.91 , 1.06) | 0.72 |  | 0.96 | (0.88 , 1.06) | 0.43 |  | 1.01 | (0.92 , 1.11) | 0.76 |  | 0.97 | (0.87 , 1.09) | 0.63 |
| I1 | 0.99 | (0.94 , 1.05) | 0.82 |  | 0.91 | (0.86 , 0.96) | <0.001* |  | 1.02 | (0.95 , 1.10) | 0.57 |  | 0.90 | (0.83 , 0.96) | 0.004* |
| I2 | 1.00 | (0.91 , 1.10) | 0.95 |  | 0.93 | (0.82 , 1.05) | 0.22 |  | 1.04 | (0.93 , 1.17) | 0.44 |  | 0.95 | (0.83 , 1.08) | 0.40 |
| **Left eye** |  |  |  |  |  |  |  |  |  |  |  |  |  |  |  |
| Retinal pigmented epithelium | | | | | | | | | | | | | | | |
| F (aka C0) | 1.04 | (0.96 , 1.13) | 0.32 |  | 0.99 | (0.84 , 1.16) | 0.88 |  | 1.08 | (0.92 , 1.27) | 0.32 |  | 1.05 | (0.88 , 1.25) | 0.60 |
| N1 | 1.06 | (0.96 , 1.18) | 0.26 |  | 0.85 | (0.67 , 1.08) | 0.17 |  | 1.21 | (0.96 , 1.53) | 0.11 |  | 1.01 | (0.76 , 1.35) | 0.93 |
| N2 | 1.06 | (0.96 , 1.17) | 0.23 |  | 0.92 | (0.70 , 1.22) | 0.58 |  | 1.06 | (0.91 , 1.24) | 0.44 |  | 0.95 | (0.69 , 1.30) | 0.76 |
| S1 | 1.07 | (0.97 , 1.17) | 0.20 |  | 0.69 | (0.52 , 0.90) | 0.01* |  | 1.13 | (0.91 , 1.39) | 0.26 |  | 0.77 | (0.55 , 1.07) | 0.11 |
| S2 | 1.09 | (0.88 , 1.34) | 0.43 |  | 0.75 | (0.55 , 1.02) | 0.06 |  | 1.11 | (0.87 , 1.43) | 0.39 |  | 0.71 | (0.49 , 1.03) | 0.07 |
| T1 | 0.90 | (0.73 , 1.12) | 0.34 |  | 0.84 | (0.65 , 1.09) | 0.19 |  | 1.09 | (0.84 , 1.41) | 0.53 |  | 1.01 | (0.74 , 1.38) | 0.96 |
| T2 | 1.08 | (0.82 , 1.42) | 0.61 |  | 0.94 | (0.67 , 1.30) | 0.70 |  | 1.20 | (0.87 , 1.67) | 0.27 |  | 1.08 | (0.73 , 1.61) | 0.70 |
| I1 | 1.06 | (0.96 , 1.17) | 0.24 |  | 0.87 | (0.67 , 1.13) | 0.30 |  | 1.09 | (0.91 , 1.31) | 0.34 |  | 0.99 | (0.77 , 1.29) | 0.96 |
| I2 | 1.00 | (0.78 , 1.28) | 0.98 |  | 0.91 | (0.67 , 1.23) | 0.52 |  | 0.99 | (0.73 , 1.33) | 0.93 |  | 0.92 | (0.63 , 1.35) | 0.68 |
| Outer retinal layer |  |  |  |  |  |  |  |  |  |  |  |  |  |  |  |
| F (aka C0) | 1.01 | (0.95 , 1.07) | 0.77 |  |  | (0.83 , 0.99) | 0.04 |  | 1.09 | (1.00 , 1.20) | 0.06 |  | 0.99 | (0.88 , 1.12) | 0.91 |
| N1 | 1.05 | (0.98 , 1.13) | 0.14 |  | 0.90 | (0.79 , 1.03) | 0.13 |  | 1.12 | (0.99 , 1.28) | 0.08 |  | 1.00 | (0.85 , 1.17) | 0.96 |
| N2 | 1.05 | (0.98 , 1.13) | 0.18 |  | 0.97 | (0.84 , 1.13) | 0.73 |  | 1.05 | (0.95 , 1.16) | 0.38 |  | 0.99 | (0.86 , 1.15) | 0.90 |
| S1 | 1.07 | (0.98 , 1.16) | 0.13 |  | 0.91 | (0.78 , 1.05) | 0.18 |  | 1.08 | (0.96 , 1.21) | 0.20 |  | 0.97 | (0.83 , 1.13) | 0.68 |
| S2 | 1.12 | (0.99 , 1.27) | 0.07 |  | 0.83 | (0.70 , 0.98) | 0.03* |  | 1.09 | (0.93 , 1.28) | 0.29 |  | 0.85 | (0.70 , 1.04) | 0.12 |
| T1 | 1.01 | (0.89 , 1.14) | 0.91 |  | 0.90 | (0.78 , 1.04) | 0.16 |  | 1.08 | (0.93 , 1.26) | 0.31 |  | 0.99 | (0.82 , 1.18) | 0.90 |
| T2 | 1.11 | (0.96 , 1.28) | 0.16 |  | 0.94 | (0.79 , 1.11) | 0.45 |  | 1.10 | (0.92 , 1.32) | 0.28 |  | 1.01 | (0.82 , 1.23) | 0.96 |
| I1 | 1.06 | (0.97 , 1.15) | 0.17 |  | 0.92 | (0.80 , 1.06) | 0.25 |  | 1.09 | (0.96 , 1.23) | 0.18 |  | 1.02 | (0.88 , 1.18) | 0.82 |
| I2 | 1.03 | (0.90 , 1.17) | 0.70 |  | 0.92 | (0.78 , 1.08) | 0.31 |  | 0.98 | (0.83 , 1.15) | 0.76 |  | 0.93 | (0.77 , 1.12) | 0.44 |
| Inner plexiform layer |  |  |  |  |  |  |  |  |  |  |  |  |  |  |  |
| F (aka C0) | 0.97 | (0.88 , 1.07) | 0.50 |  | 0.84 | (0.74 , 0.96) | 0.01* |  | 0.97 | (0.86 , 1.09) | 0.59 |  | 0.84 | (0.72 , 0.99) | 0.04* |
| N1 | 0.94 | (0.87 , 1.02) | 0.11 |  | 0.87 | (0.80 , 0.95) | 0.002* |  | 0.99 | (0.91 , 1.09) | 0.90 |  | 0.90 | (0.82 , 1.00) | 0.04* |
| N2 | 0.98 | (0.89 , 1.08) | 0.65 |  | 1.00 | (0.89 , 1.12) | 0.94 |  | 1.04 | (0.93 , 1.16) | 0.53 |  | 1.03 | (0.92 , 1.17) | 0.58 |
| S1 | 0.93 | (0.86 , 1.02) | 0.11 |  | 0.85 | (0.77 , 0.94) | 0.001* |  | 0.99 | (0.89 , 1.10) | 0.81 |  | 0.88 | (0.78 , 0.99) | 0.03* |
| S2 | 1.01 | (0.90 , 1.15) | 0.82 |  | 0.94 | (0.81 , 1.08) | 0.39 |  | 1.13 | (0.98 , 1.31) | 0.10 |  | 0.98 | (0.84 , 1.16) | 0.85 |
| T1 | 0.96 | (0.89 , 1.04) | 0.36 |  | 0.84 | (0.76 , 0.92) | <0.001* |  | 1.03 | (0.93 , 1.13) | 0.61 |  | 0.89 | (0.80 , 0.99) | 0.03* |
| T2 | 0.97 | (0.87 , 1.08) | 0.54 |  | 0.91 | (0.80 , 1.03) | 0.15 |  | 1.05 | (0.93 , 1.19) | 0.41 |  | 0.98 | (0.85 , 1.12) | 0.72 |
| I1 | 0.95 | (0.87 , 1.03) | 0.22 |  | 0.81 | (0.74 , 0.89) | <0.001* |  | 1.02 | (0.92 , 1.14) | 0.66 |  | 0.82 | (0.73 , 0.93) | 0.002* |
| I2 | 1.06 | (0.94 , 1.18) | 0.34 |  | 0.98 | (0.86 , 1.13) | 0.80 |  | 1.10 | (0.96 , 1.25) | 0.17 |  | 1.03 | (0.88 , 1.20) | 0.73 |
| Full retinal thickness |  |  |  |  |  |  |  |  |  |  |  |  |  |  |  |
| F (aka C0) | 1.00 | (0.99 , 1.01) | 0.76 |  | 0.98 | (0.96 , 1.00) | 0.03* |  | 1.00 | (0.99 , 1.01) | 0.75 |  | 0.98 | (0.96 , 1.00) | 0.09 |
| N1 | 0.99 | (0.98 , 1.01) | 0.47 |  | 0.96 | (0.94 , 0.98) | <0.001* |  | 1.00 | (0.98 , 1.01) | 0.70 |  | 0.98 | (0.95 , 1.00) | 0.03* |
| N2 | 1.00 | (0.99 , 1.02) | 0.86 |  | 0.98 | (0.96 , 1.00) | 0.07 |  | 1.00 | (0.99 , 1.02) | 0.83 |  | 0.99 | (0.96 , 1.01) | 0.28 |
| S1 | 0.99 | (0.97 , 1.01) | 0.36 |  | 0.96 | (0.94 , 0.98) | <0.001* |  | 1.00 | (0.98 , 1.01) | 0.69 |  | 0.97 | (0.95 , 0.99) | 0.01* |
| S2 | 1.00 | (0.98 , 1.02) | 0.92 |  | 0.97 | (0.94 , 0.99) | 0.02* |  | 1.01 | (0.99 , 1.03) | 0.52 |  | 0.98 | (0.95 , 1.01) | 0.16 |
| T1 | 0.99 | (0.97 , 1.01) | 0.35 |  | 0.96 | (0.94 , 0.98) | <0.001* |  | 1.00 | (0.98 , 1.02) | 0.83 |  | 0.98 | (0.96 , 1.00) | 0.08 |
| T2 | 0.99 | (0.98 , 1.01) | 0.60 |  | 1.00 | (0.98 , 1.02) | 0.87 |  | 1.00 | (0.98 , 1.02) | 0.74 |  | 1.00 | (0.99 , 1.02) | 0.71 |
| I1 | 0.99 | (0.97 , 1.01) | 0.32 |  | 0.95 | (0.93 , 0.97) | <0.001* |  | 1.00 | (0.98 , 1.01) | 0.73 |  | 0.97 | (0.94 , 0.99) | 0.01* |
| I2 | 1.00 | (0.98 , 1.02) | 0.93 |  | 0.99 | (0.96 , 1.01) | 0.36 |  | 1.00 | (0.98 , 1.02) | 0.83 |  | 1.00 | (0.97 , 1.02) | 0.81 |
| Inner retinal layer |  |  |  |  |  |  |  |  |  |  |  |  |  |  |  |
| F (aka C0) | 1.00 | (0.99 , 1.01) | 0.78 |  | 0.99 | (0.97 , 1.01) | 0.19 |  | 0.99 | (0.98 , 1.01) | 0.45 |  | 0.99 | (0.97 , 1.00) | 0.12 |
| N1 | 0.99 | (0.97 , 1.01) | 0.21 |  | 0.97 | (0.95 , 0.99) | 0.002* |  | 0.99 | (0.97 , 1.01) | 0.32 |  | 0.98 | (0.95 , 1.00) | 0.03* |
| N2 | 1.00 | (0.98 , 1.02) | 0.81 |  | 0.98 | (0.96 , 1.00) | 0.08 |  | 1.00 | (0.98 , 1.02) | 0.97 |  | 0.99 | (0.96 , 1.01) | 0.3* |
| S1 | 0.99 | (0.97 , 1.01) | 0.20 |  | 0.96 | (0.94 , 0.98) | <0.001* |  | 0.99 | (0.98 , 1.01) | 0.47 |  | 0.97 | (0.95 , 0.99) | 0.01* |
| S2 | 1.00 | (0.98 , 1.02) | 0.71 |  | 0.97 | (0.95 , 1.00) | 0.05 |  | 1.01 | (0.98 , 1.03) | 0.61 |  | 0.98 | (0.96 , 1.01) | 0.25 |
| T1 | 0.99 | (0.97 , 1.01) | 0.34 |  | 0.97 | (0.95 , 0.99) | 0.002* |  | 1.00 | (0.98 , 1.01) | 0.71 |  | 0.98 | (0.96 , 1.00) | 0.09 |
| T2 | 0.99 | (0.97 , 1.01) | 0.53 |  | 1.00 | (0.98 , 1.02) | 0.99 |  | 1.00 | (0.98 , 1.02) | 0.83 |  | 1.00 | (0.99 , 1.02) | 0.71 |
| I1 | 0.99 | (0.97 , 1.01) | 0.19 |  | 0.95 | (0.93 , 0.98) | <0.001* |  | 0.99 | (0.97 , 1.01) | 0.47 |  | 0.97 | (0.94 , 0.99) | 0.01* |
| I2 | 1.00 | (0.98 , 1.02) | 0.93 |  | 0.99 | (0.97 , 1.02) | 0.53 |  | 1.00 | (0.98 , 1.02) | 0.78 |  | 1.00 | (0.97 , 1.02) | 0.94 |
| Nerve fibre layer |  |  |  |  |  |  |  |  |  |  |  |  |  |  |  |
| F (aka C0) | 0.98 | (0.87 , 1.11) | 0.77 |  | 0.98 | (0.84 , 1.14) | 0.79 |  | 0.98 | (0.84 , 1.13) | 0.77 |  | 0.98 | (0.84 , 1.14) | 0.78 |
| N1 | 1.02 | (0.93 , 1.12) | 0.62 |  | 1.02 | (0.91 , 1.14) | 0.71 |  | 1.01 | (0.90 , 1.13) | 0.93 |  | 1.00 | (0.90 , 1.11) | 0.95 |
| N2 | 1.03 | (0.99 , 1.07) | 0.13 |  | 0.97 | (0.93 , 1.02) | 0.29 |  | 1.03 | (0.98 , 1.08) | 0.21 |  | 0.98 | (0.93 , 1.03) | 0.48 |
| S1 | 1.06 | (0.98 , 1.15) | 0.18 |  | 0.97 | (0.87 , 1.08) | 0.58 |  | 1.03 | (0.94 , 1.13) | 0.52 |  | 0.99 | (0.89 , 1.10) | 0.85 |
| S2 | 1.02 | (0.97 , 1.08) | 0.38 |  | 0.98 | (0.92 , 1.04) | 0.43 |  | 1.02 | (0.97 , 1.08) | 0.45 |  | 0.98 | (0.92 , 1.05) | 0.57 |
| T1 | 1.09 | (0.92 , 1.29) | 0.32 |  | 1.21 | (1.03 , 1.43) | 0.02 |  | 0.95 | (0.77 , 1.17) | 0.63 |  | 1.14 | (0.96 , 1.36) | 0.14 |
| T2 | 1.12 | (0.96 , 1.31) | 0.14 |  | 1.16 | (0.99 , 1.36) | 0.06 |  | 1.02 | (0.87 , 1.19) | 0.83 |  | 1.06 | (0.95 , 1.18) | 0.33 |
| I1 | 1.00 | (0.91 , 1.09) | 0.92 |  | 0.99 | (0.89 , 1.10) | 0.84 |  | 1.02 | (0.92 , 1.13) | 0.70 |  | 1.00 | (0.90 , 1.11) | 0.94 |
| I2 | 1.01 | (0.96 , 1.05) | 0.77 |  | 1.00 | (0.95 , 1.06) | 1.00 |  | 1.01 | (0.96 , 1.07) | 0.65 |  | 1.00 | (0.94 , 1.06) | 0.92 |
| Outer plexiform layer | | | | | | | | | | | | | | | |
| F (aka C0) | 1.05 | (0.99 , 1.12) | 0.09 |  | 0.99 | (0.92 , 1.08) | 0.88 |  | 1.05 | (0.97 , 1.13) | 0.20 |  | 0.99 | (0.90 , 1.08) | 0.82 |
| N1 | 1.01 | (0.96 , 1.07) | 0.64 |  | 1.01 | (0.95 , 1.08) | 0.64 |  | 1.00 | (0.94 , 1.07) | 0.89 |  | 1.00 | (0.93 , 1.08) | 0.94 |
| N2 | 1.06 | (0.93 , 1.20) | 0.38 |  | 0.97 | (0.82 , 1.15) | 0.74 |  | 1.06 | (0.89 , 1.26) | 0.51 |  | 1.00 | (0.82 , 1.22) | 1.00 |
| S1 | 0.99 | (0.95 , 1.03) | 0.63 |  | 0.92 | (0.87 , 0.99) | 0.02* |  | 1.00 | (0.95 , 1.06) | 0.93 |  | 0.93 | (0.87 , 0.99) | 0.03 |
| S2 | 0.94 | (0.84 , 1.05) | 0.26 |  | 0.74 | (0.62 , 0.89) | 0.001* |  | 1.02 | (0.89 , 1.17) | 0.80 |  | 0.82 | (0.68 , 0.99) | 0.04 |
| T1 | 1.07 | (0.99 , 1.15) | 0.08 |  | 0.95 | (0.86 , 1.05) | 0.32 |  | 1.11 | (1.02 , 1.22) | 0.02* |  | 1.01 | (0.90 , 1.13) | 0.84 |
| T2 | 1.08 | (0.93 , 1.25) | 0.30 |  | 0.87 | (0.71 , 1.06) | 0.17 |  | 1.22 | (1.01 , 1.46) | 0.04* |  | 1.02 | (0.82 , 1.26) | 0.87 |
| I1 | 1.05 | (1.00 , 1.11) | 0.06 |  | 1.08 | (1.01 , 1.14) | 0.02 |  | 1.04 | (0.97 , 1.12) | 0.24 |  | 1.08 | (1.01 , 1.16) | 0.03* |
| I2 | 1.11 | (0.97 , 1.27) | 0.15 |  | 1.02 | (0.85 , 1.23) | 0.81 |  | 1.13 | (0.91 , 1.40) | 0.25 |  | 1.08 | (0.86 , 1.35) | 0.53 |
| Inner nuclear layer |  |  |  |  |  |  |  |  |  |  |  |  |  |  |  |
| F (aka C0) | 1.01 | (0.96 , 1.07) | 0.58 |  | 1.07 | (1.02 , 1.12) | 0.01* |  | 0.98 | (0.92 , 1.05) | 0.61 |  | 1.04 | (0.98 , 1.11) | 0.23 |
| N1 | 0.93 | (0.85 , 1.01) | 0.08 |  | 0.92 | (0.83 , 1.02) | 0.10 |  | 0.90 | (0.81 , 1.01) | 0.07 |  | 0.90 | (0.79 , 1.02) | 0.11 |
| N2 | 0.97 | (0.87 , 1.08) | 0.59 |  | 0.98 | (0.86 , 1.12) | 0.75 |  | 1.01 | (0.9 , 1.15) | 0.83 |  | 1.05 | (0.91 , 1.20) | 0.51 |
| S1 | 0.91 | (0.84 , 0.98) | 0.02* |  | 0.84 | (0.76 , 0.92) | <0.001* |  | 0.92 | (0.84 , 1.01) | 0.09 |  | 0.88 | (0.79 , 0.97) | 0.01 |
| S2 | 0.95 | (0.84 , 1.07) | 0.40 |  | 0.90 | (0.78 , 1.05) | 0.18 |  | 1.04 | (0.90 , 1.21) | 0.60 |  | 0.96 | (0.82 , 1.14) | 0.67 |
| T1 | 0.95 | (0.87 , 1.04) | 0.26 |  | 0.87 | (0.79 , 0.97) | 0.01* |  | 0.93 | (0.83 , 1.03) | 0.17 |  | 0.88 | (0.78 , 0.99) | 0.03 |
| T2 | 0.94 | (0.83 , 1.06) | 0.32 |  | 1.03 | (0.92 , 1.15) | 0.57 |  | 1.01 | (0.88 , 1.16) | 0.85 |  | 1.07 | (0.95 , 1.21) | 0.28 |
| I1 | 0.95 | (0.87 , 1.03) | 0.24 |  | 0.88 | (0.79 , 0.98) | 0.02* |  | 0.93 | (0.84 , 1.03) | 0.16 |  | 0.89 | (0.79 , 1.00) | 0.06 |
| I2 | 1.00 | (0.89 , 1.12) | 0.98 |  | 1.08 | (0.97 , 1.2) | 0.18 |  | 1.02 | (0.89 , 1.17) | 0.74 |  | 1.11 | (0.97 , 1.27) | 0.12 |
| Outer nuclear layer |  |  |  |  |  |  |  |  |  |  |  |  |  |  |  |
| F (aka C0) | 1.00 | (0.98 , 1.02) | 0.87 |  | 0.93 | (0.90 , 0.97) | <0.001* |  | 0.99 | (0.97 , 1.01) | 0.40 |  | 0.93 | (0.89 , 0.97) | 0.001* |
| N1 | 0.99 | (0.97 , 1.02) | 0.52 |  | 0.96 | (0.93 , 0.99) | 0.02* |  | 0.99 | (0.96 , 1.02) | 0.35 |  | 0.97 | (0.93 , 1.01) | 0.10 |
| N2 | 0.97 | (0.93 , 1.01) | 0.18 |  | 0.95 | (0.90 , 1.00) | 0.07 |  | 0.97 | (0.93 , 1.02) | 0.27 |  | 0.96 | (0.91 , 1.02) | 0.20 |
| S1 | 0.99 | (0.97 , 1.02) | 0.63 |  | 0.99 | (0.96 , 1.02) | 0.44 |  | 0.99 | (0.97 , 1.02) | 0.45 |  | 0.99 | (0.96 , 1.03) | 0.61 |
| S2 | 0.99 | (0.95 , 1.03) | 0.52 |  | 0.98 | (0.93 , 1.03) | 0.41 |  | 0.99 | (0.95 , 1.03) | 0.53 |  | 0.98 | (0.92 , 1.04) | 0.54 |
| T1 | 0.98 | (0.95 , 1.01) | 0.21 |  | 0.98 | (0.94 , 1.02) | 0.26 |  | 0.98 | (0.94 , 1.01) | 0.20 |  | 0.98 | (0.95 , 1.02) | 0.42 |
| T2 | 0.97 | (0.93 , 1.02) | 0.28 |  | 1.01 | (0.97 , 1.04) | 0.71 |  | 0.98 | (0.93 , 1.03) | 0.34 |  | 1.01 | (0.97 , 1.04) | 0.73 |
| I1 | 0.97 | (0.94 , 1.00) | 0.06 |  | 0.94 | (0.90 , 0.97) | <0.001* |  | 0.96 | (0.92 , 1.00) | 0.07 |  | 0.94 | (0.89 , 0.98) | 0.01* |
| I2 | 0.97 | (0.93 , 1.02) | 0.28 |  | 0.97 | (0.91 , 1.03) | 0.30 |  | 0.98 | (0.94 , 1.03) | 0.44 |  | 0.98 | (0.92 , 1.04) | 0.50 |
| Ganglion cell layer |  |  |  |  |  |  |  |  |  |  |  |  |  |  |  |
| F (aka C0) | 1.02 | (0.95 , 1.09) | 0.57 |  | 1.02 | (0.95 , 1.11) | 0.57 |  | 1.03 | (0.94 , 1.12) | 0.54 |  | 1.02 | (0.93 , 1.12) | 0.65 |
| N1 | 0.97 | (0.92 , 1.02) | 0.26 |  | 0.93 | (0.87 , 0.98) | 0.01* |  | 1.00 | (0.94 , 1.07) | 0.98 |  | 0.94 | (0.87 , 1.01) | 0.08 |
| N2 | 0.98 | (0.9 , 1.06) | 0.62 |  | 0.94 | (0.85 , 1.04) | 0.24 |  | 1.03 | (0.93 , 1.14) | 0.56 |  | 0.97 | (0.86 , 1.08) | 0.55 |
| S1 | 0.98 | (0.92 , 1.04) | 0.53 |  | 0.92 | (0.87 , 0.99) | 0.02* |  | 1.01 | (0.94 , 1.09) | 0.75 |  | 0.95 | (0.88 , 1.03) | 0.18 |
| S2 | 0.99 | (0.90 , 1.09) | 0.81 |  | 0.96 | (0.86 , 1.08) | 0.50 |  | 1.07 | (0.95 , 1.19) | 0.26 |  | 0.99 | (0.87 , 1.12) | 0.86 |
| T1 | 0.98 | (0.93 , 1.03) | 0.50 |  | 0.91 | (0.86 , 0.96) | 0.001* |  | 1.01 | (0.95 , 1.07) | 0.74 |  | 0.93 | (0.87 , 0.99) | 0.02* |
| T2 | 0.97 | (0.90 , 1.05) | 0.43 |  | 0.92 | (0.84 , 1.01) | 0.09 |  | 1.04 | (0.95 , 1.13) | 0.45 |  | 0.95 | (0.86 , 1.06) | 0.38 |
| I1 | 0.98 | (0.93 , 1.04) | 0.51 |  | 0.89 | (0.84 , 0.95) | <0.001* |  | 1.03 | (0.96 , 1.10) | 0.47 |  | 0.90 | (0.84 , 0.97) | 0.004* |
| I2 | 1.03 | (0.93 , 1.13) | 0.57 |  | 0.93 | (0.83 , 1.04) | 0.22 |  | 1.08 | (0.97 , 1.21) | 0.17 |  | 0.97 | (0.85 , 1.11) | 0.71 |

ETDRS, Early Treatment Diabetic Retinopathy Study; OR, odds ratio; SD, standard deviation; CI, Confidence interval; F, fovea; S1, superior segment 1; N1, nasal segment 1; I1, inferior segment 1; T1, temporal segment 1.*significant values. Adjustment for age, mean arterial blood pressure, diabetes status, low-density lipoprotein, body mass index, and sex.

**Table S2:** Regression coefficients (β) between thickness (µm) of the retinal layers with eGFR (ml/min/1.73m^2^) for ETDRS grid annulus 1.

|  | **Unadjusted model** | | |  | **Adjusted model** | | |
| --- | --- | --- | --- | --- | --- | --- | --- |
| ETDRS segment | β | 95% CI | p |  | β | 95% CI | p |
| **Right eye** |  |  |  |  |  |  |  |
| Full retinal thickness | | | | | | | |
| F (aka C0) | 0.20 | (0.07 , 0.34) | <0.001* |  | 0.19 | (0.04 , 0.34) | 0.01* |
| N1 | 0.32 | (0.16 , 0.48) | <0.001* |  | 0.22 | (0.05 , 0.39) | 0.01* |
| N2 | 0.16 | (-0.02 , 0.34) | 0.08 |  | 0.09 | (-0.09 , 0.26) | 0.34 |
| S1 | 0.27 | (0.11 , 0.43) | <0.001* |  | 0.18 | (0.01 , 0.36) | 0.04* |
| S2 | 0.08 | (-0.12 , 0.29) | 0.42 |  | 0.02 | (-0.18 , 0.23) | 0.81 |
| T1 | 0.15 | (0.00 , 0.29) | 0.05* |  | 0.07 | (-0.08 , 0.21) | 0.35 |
| T2 | -0.04 | (-0.20 , 0.11) | 0.59 |  | -0.08 | (-0.22 , 0.06) | 0.27 |
| I1 | 0.33 | (0.16 , 0.49) | <0.001* |  | 0.22 | (0.05 , 0.39) | 0.01* |
| I2 | 0.13 | (-0.06 , 0.32) | 0.19 |  | 0.04 | (-0.15 , 0.23) | 0.66 |
| Inner Retinal Layer | | | | | | | |
| F (aka C0) | 0.17 | (0.04 , 0.31) | 0.01* |  | 0.19 | (0.04 , 0.35) | 0.02* |
| N1 | 0.31 | (0.15 , 0.48) | <0.001* |  | 0.23 | (0.05 , 0.41) | 0.01* |
| N2 | 0.15 | (-0.03 , 0.33) | 0.11 |  | 0.08 | (-0.10 , 0.26) | 0.38 |
| S1 | 0.27 | (0.11 , 0.44) | <0.001* |  | 0.19 | (0.01 , 0.37) | 0.03* |
| S2 | 0.07 | (-0.14 , 0.28) | 0.52 |  | 0.00 | (-0.20 , 0.21) | 0.97 |
| T1 | 0.14 | (-0.01 , 0.29) | 0.07 |  | 0.07 | (-0.08 , 0.22) | 0.37 |
| T2 | -0.05 | (-0.20 , 0.11) | 0.56 |  | -0.09 | (-0.24 , 0.05) | 0.22 |
| I1 | 0.32 | (0.15 , 0.48) | <0.001* |  | 0.23 | (0.05 , 0.40) | 0.01* |
| I2 | 0.10 | (-0.10 , 0.29) | 0.34 |  | 0.01 | (-0.18 , 0.21) | 0.88 |
| Outer retinal layer | | | | | | | |
| F (aka C0) | 0.41 | (-0.11 , 0.92) | 0.12 |  | -0.02 | (-0.54 , 0.50) | 0.93 |
| N1 | 0.59 | (-0.33 , 1.52) | 0.21 |  | 0.04 | (-0.93 , 1.00) | 0.94 |
| N2 | 0.70 | (-0.42 , 1.82) | 0.22 |  | 0.57 | (-0.68 , 1.83) | 0.37 |
| S1 | 0.37 | (-0.68 , 1.43) | 0.48 |  | 0.01 | (-1.08 , 1.09) | 0.99 |
| S2 | 0.61 | (-0.53 , 1.76) | 0.29 |  | 0.80 | (-0.43 , 2.02) | 0.20 |
| T1 | 0.54 | (-0.48 , 1.57) | 0.30 |  | -0.11 | (-1.19 , 0.97) | 0.83 |
| T2 | -0.03 | (-0.90 , 0.83) | 0.94 |  | 0.15 | (-0.73 , 1.03) | 0.74 |
| I1 | 0.46 | (-0.56 , 1.48) | 0.38 |  | -0.02 | (-1.13 , 1.09) | 0.97 |
| I2 | 1.01 | (-0.11 , 2.12) | 0.08 |  | 0.94 | (-0.29 , 2.16) | 0.13 |
| Nerve fibre layer | | | | | | | |
| F (aka C0) | 1.57 | (0.28 , 2.85) | 0.02* |  | 0.86 | (-0.49 , 2.22) | 0.21 |
| N1 | 0.23 | (-0.94 , 1.39) | 0.70 |  | 0.24 | (-0.93 , 1.40) | 0.69 |
| N2 | 0.03 | (-0.35 , 0.41) | 0.88 |  | -0.04 | (-0.42 , 0.34) | 0.84 |
| S1 | -0.65 | (-1.41 , 0.11) | 0.09 |  | -0.70 | (-1.44 , 0.05) | 0.07 |
| S2 | -0.31 | (-0.79 , 0.18) | 0.21 |  | -0.35 | (-0.83 , 0.14) | 0.16 |
| T1 | -0.82 | (-1.51 , -0.13) | 0.02* |  | -0.69 | (-1.33 , -0.04) | 0.04* |
| T2 | -1.35 | (-2.23 , -0.46) | <0.001* |  | -0.97 | (-1.79 , -0.14) | 0.02* |
| I1 | 0.39 | (-0.40 , 1.18) | 0.33 |  | 0.17 | (-0.62 , 0.96) | 0.68 |
| I2 | 0.16 | (-0.26 , 0.58) | 0.45 |  | 0.14 | (-0.27 , 0.56) | 0.49 |
| Ganglion cell layer | | | | | | | |
| F (aka C0) | 0.26 | (-0.37 , 0.88) | 0.42 |  | 0.00 | (-0.65 , 0.66) | 0.99 |
| N1 | 0.56 | (0.13 , 1.00) | 0.01* |  | 0.27 | (-0.21 , 0.76) | 0.27 |
| N2 | 0.51 | (-0.22 , 1.24) | 0.17 |  | 0.28 | (-0.49 , 1.05) | 0.47 |
| S1 | 0.52 | (0.07 , 0.98) | 0.03* |  | 0.34 | (-0.16 , 0.84) | 0.18 |
| S2 | 0.42 | (-0.48 , 1.33) | 0.36 |  | 0.30 | (-0.66 , 1.26) | 0.53 |
| T1 | 0.70 | (0.23 , 1.17) | <0.001* |  | 0.55 | (0.03 , 1.08) | 0.04* |
| T2 | 0.21 | (-0.51 , 0.93) | 0.56 |  | -0.13 | (-0.88 , 0.61) | 0.72 |
| I1 | 0.75 | (0.29 , 1.21) | <0.001* |  | 0.57 | (0.07 , 1.08) | 0.03* |
| I2 | 0.49 | (-0.40 , 1.37) | 0.28 |  | 0.11 | (-0.78 , 1.01) | 0.80 |
| Inner plexiform layer | | | | | | | |
| F (aka C0) | 1.01 | (0.19 , 1.84) | 0.02* |  | 0.65 | (-0.25 , 1.55) | 0.16 |
| N1 | 0.99 | (0.37 , 1.60) | <0.001* |  | 0.51 | (-0.19 , 1.20) | 0.15 |
| N2 | 0.32 | (-0.65 , 1.28) | 0.52 |  | -0.04 | (-1.04 , 0.95) | 0.93 |
| S1 | 0.93 | (0.26 , 1.59) | 0.01* |  | 0.56 | (-0.15 , 1.28) | 0.12 |
| S2 | 0.33 | (-0.83 , 1.49) | 0.58 |  | 0.20 | (-0.98 , 1.38) | 0.74 |
| T1 | 1.04 | (0.37 , 1.71) | <0.001* |  | 0.72 | (-0.04 , 1.48) | 0.06 |
| T2 | 0.47 | (-0.51 , 1.45) | 0.34 |  | -0.05 | (-1.05 , 0.94) | 0.91 |
| I1 | 1.36 | (0.69 , 2.02) | <0.001* |  | 0.90 | (0.16 , 1.64) | 0.02* |
| I2 | 0.63 | (-0.47 , 1.73) | 0.26 |  | 0.10 | (-1.02 , 1.22) | 0.86 |
| Inner nuclear layer | | | | | | | |
| F (aka C0) | -0.07 | (-0.58 , 0.45) | 0.80 |  | 0.18 | (-0.40 , 0.77) | 0.54 |
| N1 | 1.09 | (0.33 , 1.85) | 0.01* |  | 0.81 | (0.00 , 1.61) | 0.05* |
| N2 | 0.50 | (-0.61 , 1.6) | 0.38 |  | 0.09 | (-0.99 , 1.16) | 0.87 |
| S1 | 1.20 | (0.50 , 1.91) | <0.001* |  | 0.84 | (0.13 , 1.55) | 0.02* |
| S2 | 0.34 | (-0.80 , 1.48) | 0.56 |  | -0.15 | (-1.28 , 0.98) | 0.80 |
| T1 | 1.40 | (0.58 , 2.23) | <0.001* |  | 1.35 | (0.48 , 2.23) | <0.001* |
| T2 | 0.51 | (-0.53 , 1.55) | 0.34 |  | -0.26 | (-1.34 , 0.81) | 0.63 |
| I1 | 0.76 | (-0.02 , 1.54) | 0.06 |  | 0.57 | (-0.21 , 1.35) | 0.15 |
| I2 | -0.24 | (-1.38 , 0.90) | 0.68 |  | -0.54 | (-1.69 , 0.62) | 0.36 |
| Outer plexiform layer | | | | | | | |
| F (aka C0) | 0.21 | (-0.36 , 0.78) | 0.47 |  | 0.03 | (-0.55 , 0.62) | 0.91 |
| N1 | -0.31 | (-0.80 , 0.18) | 0.21 |  | -0.26 | (-0.73 , 0.21) | 0.28 |
| N2 | 0.08 | (-1.07 , 1.24) | 0.89 |  | 0.02 | (-1.12 , 1.16) | 0.97 |
| S1 | 0.59 | (0.13 , 1.06) | 0.01* |  | 0.23 | (-0.26 , 0.72) | 0.36 |
| S2 | 0.97 | (-0.18 , 2.12) | 0.10 |  | 0.38 | (-0.79 , 1.56) | 0.52 |
| T1 | 0.58 | (-0.27 , 1.43) | 0.18 |  | 0.03 | (-0.83 , 0.88) | 0.95 |
| T2 | 0.89 | (-0.70 , 2.49) | 0.27 |  | -0.12 | (-1.73 , 1.50) | 0.89 |
| I1 | -0.39 | (-0.96 , 0.18) | 0.18 |  | -0.28 | (-0.83 , 0.27) | 0.31 |
| I2 | -0.66 | (-2.04 , 0.72) | 0.35 |  | -0.41 | (-1.73 , 0.92) | 0.55 |
| Outer nuclear layer | | | |  |  |  |  |
| F (aka C0) | 0.47 | (0.18 , 0.75) | <0.001* |  | 0.52 | (0.24 , 0.81) | <0.001* |
| N1 | 0.38 | (0.10 , 0.67) | 0.01* |  | 0.40 | (0.11 , 0.68) | 0.01* |
| N2 | 0.44 | (0.04 , 0.84) | 0.03* |  | 0.38 | (-0.01 , 0.77) | 0.06 |
| S1 | 0.10 | (-0.20 , 0.41) | 0.50 |  | 0.21 | (-0.10 , 0.51) | 0.19 |
| S2 | 0.21 | (-0.23 , 0.64) | 0.35 |  | 0.17 | (-0.27 , 0.61) | 0.45 |
| T1 | 0.00 | (-0.28 , 0.29) | 0.99 |  | 0.00 | (-0.27 , 0.27) | 0.98 |
| T2 | -0.15 | (-0.43 , 0.13) | 0.28 |  | -0.17 | (-0.43 , 0.09) | 0.21 |
| I1 | 0.43 | (0.10 , 0.75) | 0.01* |  | 0.31 | (-0.01 , 0.63) | 0.06 |
| I2 | 0.22 | (-0.24 , 0.68) | 0.35 |  | 0.01 | (-0.44 , 0.46) | 0.97 |
| Retinal pigmented epithelium | | | |  |  |  |  |
| F (aka C0) | -0.19 | (-1.56 , 1.18) | 0.79 |  | -0.71 | (-2.04 , 0.62) | 0.29 |
| N1 | 1.77 | (-0.03 , 3.57) | 0.05 |  | 0.52 | (-1.37 , 2.41) | 0.59 |
| N2 | 1.99 | (0.06 , 3.91) | 0.04* |  | 1.46 | (-0.60 , 3.53) | 0.16 |
| S1 | 1.44 | (-0.48 , 3.37) | 0.14 |  | 0.60 | (-1.42 , 2.63) | 0.56 |
| S2 | 1.37 | (-0.86 , 3.60) | 0.23 |  | 1.26 | (-1.01 , 3.53) | 0.27 |
| T1 | 1.32 | (-0.69 , 3.33) | 0.20 |  | 0.30 | (-1.80 , 2.40) | 0.78 |
| T2 | -0.02 | (-1.18 , 1.14) | 0.97 |  | 0.29 | (-0.79 , 1.38) | 0.59 |
| I1 | 1.90 | (0.07 , 3.73) | 0.04* |  | 1.04 | (-0.79 , 2.86) | 0.26 |
| I2 | 2.04 | (-0.15 , 4.22) | 0.07 |  | 1.63 | (-0.60 , 3.86) | 0.15 |
| **Left eye** | | | | | | | |
| Full retinal thickness | | | | | | | |
| F (aka C0) | 0.08 | (-0.03 , 0.18) | 0.16 |  | 0.07 | (-0.03 , 0.17) | 0.18 |
| N1 | 0.19 | (0.06 , 0.33) | 0.01* |  | 0.12 | (-0.01 , 0.25) | 0.07 |
| N2 | 0.07 | (-0.08 , 0.22) | 0.36 |  | 0.02 | (-0.13 , 0.16) | 0.81 |
| S1 | 0.25 | (0.10 , 0.39) | <0.001* |  | 0.14 | (0.01 , 0.28) | 0.04* |
| S2 | 0.16 | (-0.03 , 0.34) | 0.10 |  | 0.05 | (-0.13 , 0.23) | 0.60 |
| T1 | 0.21 | (0.06 , 0.35) | 0.01* |  | 0.11 | (-0.03 , 0.25) | 0.12 |
| T2 | 0.03 | (-0.13 , 0.18) | 0.75 |  | -0.05 | (-0.2 , 0.10) | 0.49 |
| I1 | 0.27 | (0.13 , 0.42) | <0.001* |  | 0.17 | (0.03 , 0.31) | 0.02* |
| I2 | 0.07 | (-0.11 , 0.25) | 0.44 |  | -0.01 | (-0.18 , 0.16) | 0.91 |
| Inner Retinal Layer | | | | | | | |
| F (aka C0) | 0.04 | (-0.07 , 0.15) | 0.47 |  | 0.06 | (-0.04 , 0.17) | 0.24 |
| N1 | 0.19 | (0.05 , 0.34) | 0.01* |  | 0.13 | (-0.01 , 0.27) | 0.07 |
| N2 | 0.08 | (-0.08 , 0.24) | 0.31 |  | 0.02 | (-0.13 , 0.17) | 0.83 |
| S1 | 0.25 | (0.10 , 0.39) | <0.001* |  | 0.15 | (0.01 , 0.29) | 0.04* |
| S2 | 0.14 | (-0.05 , 0.33) | 0.15 |  | 0.03 | (-0.16 , 0.21) | 0.77 |
| T1 | 0.19 | (0.04 , 0.34) | 0.01* |  | 0.11 | (-0.03 , 0.24) | 0.14 |
| T2 | 0.02 | (-0.15 , 0.18) | 0.83 |  | -0.06 | (-0.21 , 0.10) | 0.47 |
| I1 | 0.27 | (0.12 , 0.42) | <0.001* |  | 0.17 | (0.02 , 0.32) | 0.02* |
| I2 | 0.05 | (-0.13 , 0.23) | 0.60 |  | -0.04 | (-0.21 , 0.14) | 0.68 |
| Outer Retinal Layer | | | | | | | |
| F (aka C0) | 0.67 | (0.06 , 1.27) | 0.03* |  | 0.06 | (-0.55 , 0.67) | 0.84 |
| N1 | 0.09 | (-0.51 , 0.69) | 0.77 |  | -0.04 | (-0.60 , 0.53) | 0.90 |
| N2 | -0.12 | (-0.84 , 0.60) | 0.75 |  | 0.06 | (-0.62 , 0.73) | 0.87 |
| S1 | 0.12 | (-0.71 , 0.95) | 0.77 |  | 0.04 | (-0.74 , 0.83) | 0.92 |
| S2 | 0.47 | (-0.72 , 1.66) | 0.44 |  | 0.91 | (-0.32 , 2.14) | 0.15 |
| T1 | 0.87 | (-0.26 , 2.01) | 0.13 |  | 0.11 | (-1.08 , 1.29) | 0.86 |
| T2 | 0.28 | (-1.08 , 1.63) | 0.69 |  | 0.25 | (-1.18 , 1.67) | 0.73 |
| I1 | 0.27 | (-0.60 , 1.13) | 0.55 |  | 0.03 | (-0.81 , 0.87) | 0.94 |
| I2 | 0.62 | (-0.63 , 1.85) | 0.33 |  | 1.02 | (-0.27 , 2.30) | 0.12 |
| Nerve fibre layer | | | | | | | |
| F (aka C0) | 0.43 | (-0.72 , 1.59) | 0.46 |  | 0.16 | (-0.96 , 1.27) | 0.78 |
| N1 | -0.39 | (-1.32 , 0.54) | 0.41 |  | -0.29 | (-1.16 , 0.57) | 0.50 |
| N2 | 0.05 | (-0.33 , 0.42) | 0.81 |  | -0.03 | (-0.39 , 0.33) | 0.88 |
| S1 | -0.20 | (-1.02 , 0.62) | 0.64 |  | -0.16 | (-0.94 , 0.62) | 0.68 |
| S2 | -0.02 | (-0.51 , 0.46) | 0.93 |  | -0.06 | (-0.53 , 0.41) | 0.79 |
| T1 | -1.79 | (-3.12 , -0.46) | 0.01* |  | -1.36 | (-2.61 , -0.11) | 0.03* |
| T2 | -1.28 | (-2.22 , -0.35) | 0.01* |  | -0.78 | (-1.64 , 0.08) | 0.08 |
| I1 | 0.08 | (-0.75 , 0.91) | 0.85 |  | -0.28 | (-1.08 , 0.52) | 0.49 |
| I2 | -0.01 | (-0.44 , 0.42) | 0.96 |  | -0.13 | (-0.55 , 0.29) | 0.54 |
| Ganglion cell layer | | | | | | | |
| F (aka C0) | -0.19 | (-0.85 , 0.47) | 0.57 |  | -0.25 | (-0.88 , 0.38) | 0.43 |
| N1 | 0.72 | (0.24 , 1.21) | <0.001* |  | 0.35 | (-0.16 , 0.86) | 0.18 |
| N2 | 0.29 | (-0.50 , 1.07) | 0.47 |  | -0.12 | (-0.92 , 0.67) | 0.76 |
| S1 | 0.64 | (0.08 , 1.19) | 0.03* |  | 0.25 | (-0.31 , 0.82) | 0.37 |
| S2 | 0.18 | (-0.74 , 1.10) | 0.70 |  | -0.30 | (-1.21 , 0.60) | 0.51 |
| T1 | 0.66 | (0.20 , 1.11) | 0.01* |  | 0.35 | (-0.12 , 0.82) | 0.14 |
| T2 | 0.54 | (-0.18 , 1.26) | 0.14 |  | -0.03 | (-0.75 , 0.69) | 0.94 |
| I1 | 0.90 | (0.42 , 1.38) | <0.001* |  | 0.53 | (0.03 , 1.02) | 0.04* |
| I2 | 0.41 | (-0.48 , 1.31) | 0.37 |  | -0.08 | (-0.97 , 0.81) | 0.85 |
| Inner plexiform layer | | | | | | | |
| F (aka C0) | 1.16 | (0.26 , 2.06) | 0.01* |  | 0.85 | (-0.08 , 1.77) | 0.07 |
| N1 | 1.25 | (0.56 , 1.95) | <0.001* |  | 0.70 | (-0.02 , 1.42) | 0.06 |
| N2 | 0.05 | (-0.86 , 0.96) | 0.92 |  | -0.43 | (-1.32 , 0.46) | 0.34 |
| S1 | 1.32 | (0.54 , 2.10) | <0.001* |  | 0.71 | (-0.12 , 1.53) | 0.09 |
| S2 | 0.34 | (-0.82 , 1.49) | 0.57 |  | -0.41 | (-1.55 , 0.73) | 0.48 |
| T1 | 1.34 | (0.61 , 2.06) | <0.001* |  | 0.69 | (-0.05 , 1.43) | 0.07 |
| T2 | 0.70 | (-0.28 , 1.67) | 0.16 |  | -0.10 | (-1.07 , 0.86) | 0.83 |
| I1 | 1.65 | (0.91 , 2.39) | <0.001* |  | 1.06 | (0.28 , 1.83) | 0.01* |
| I2 | 0.02 | (-1.06 , 1.09) | 0.98 |  | -0.46 | (-1.52 , 0.60) | 0.40 |
| Inner nuclear layer | | | | | | | |
| F (aka C0) | -0.51 | (-0.94 , -0.08) | 0.02* |  | -0.26 | (-0.69 , 0.17) | 0.24 |
| N1 | 0.97 | (0.19 , 1.75) | 0.02* |  | 0.70 | (-0.10 , 1.50) | 0.09 |
| N2 | 0.72 | (-0.31 , 1.75) | 0.17 |  | 0.04 | (-0.96 , 1.03) | 0.94 |
| S1 | 1.57 | (0.92 , 2.22) | <0.001* |  | 1.05 | (0.41 , 1.70) | <0.001* |
| S2 | 1.49 | (0.39 , 2.58) | 0.01* |  | 0.76 | (-0.36 , 1.87) | 0.18 |
| T1 | 1.05 | (0.24 , 1.86) | 0.01* |  | 0.94 | (0.15 , 1.73) | 0.02* |
| T2 | 0.26 | (-0.73 , 1.25) | 0.61 |  | -0.37 | (-1.35 , 0.60) | 0.45 |
| I1 | 0.97 | (0.20 , 1.73) | 0.01* |  | 0.76 | (0.01 , 1.51) | 0.05* |
| I2 | -0.13 | (-1.13 , 0.87) | 0.80 |  | -0.35 | (-1.32 , 0.61) | 0.47 |
| Outer plexiform layer | | | | | | | |
| F (aka C0) | -0.12 | (-0.72 , 0.49) | 0.71 |  | -0.10 | (-0.68 , 0.49) | 0.75 |
| N1 | -0.10 | (-0.62 , 0.43) | 0.71 |  | -0.06 | (-0.55 , 0.44) | 0.83 |
| N2 | -0.01 | (-1.28 , 1.26) | 0.99 |  | -0.03 | (-1.25 , 1.20) | 0.97 |
| S1 | 0.50 | (0.11 , 0.90) | 0.01* |  | 0.33 | (-0.06 , 0.72) | 0.09 |
| S2 | 1.90 | (0.93 , 2.88) | <0.001* |  | 1.20 | (0.19 , 2.21) | 0.02* |
| T1 | 0.10 | (-0.63 , 0.84) | 0.79 |  | -0.15 | (-0.85 , 0.54) | 0.66 |
| T2 | 0.72 | (-0.73 , 2.16) | 0.33 |  | -0.10 | (-1.51 , 1.31) | 0.89 |
| I1 | -0.80 | (-1.34 , -0.26) | <0.001* |  | -0.62 | (-1.16 , -0.08) | 0.02* |
| I2 | -0.61 | (-2.03 , 0.80) | 0.39 |  | -0.61 | (-2.2 , 0.97) | 0.45 |
| Outer nuclear layer | | | | | | | |
| F (aka C0) | 0.14 | (-0.03 , 0.30) | 0.10 |  | 0.16 | (0.01 , 0.31) | 0.04* |
| N1 | 0.12 | (-0.08 , 0.31) | 0.24 |  | 0.12 | (-0.06 , 0.31) | 0.19 |
| N2 | 0.12 | (-0.13 , 0.36) | 0.34 |  | 0.09 | (-0.14 , 0.32) | 0.44 |
| S1 | 0.02 | (-0.19 , 0.23) | 0.83 |  | 0.04 | (-0.16 , 0.23) | 0.71 |
| S2 | 0.04 | (-0.30 , 0.39) | 0.80 |  | 0.00 | (-0.32 , 0.32) | 1.00 |
| T1 | 0.14 | (-0.10 , 0.38) | 0.26 |  | 0.11 | (-0.11 , 0.33) | 0.33 |
| T2 | -0.01 | (-0.31 , 0.30) | 0.97 |  | -0.06 | (-0.34 , 0.22) | 0.68 |
| I1 | 0.33 | (0.10 , 0.57) | 0.01* |  | 0.29 | (0.06 , 0.52) | 0.01* |
| I2 | 0.14 | (-0.19 , 0.47) | 0.41 |  | 0.06 | (-0.25 , 0.37) | 0.71 |
| Retinal pigmented epithelium | | | | | | | |
| F (aka C0) | 0.00 | (-0.91 , 0.92) | 0.99 |  | 0.03 | (-0.80 , 0.87) | 0.94 |
| N1 | -0.02 | (-0.66 , 0.63) | 0.96 |  | 0.01 | (-0.57 , 0.59) | 0.96 |
| N2 | -0.12 | (-0.94 , 0.70) | 0.77 |  | 0.10 | (-0.65 , 0.84) | 0.80 |
| S1 | 0.36 | (-0.59 , 1.31) | 0.45 |  | 0.22 | (-0.65 , 1.10) | 0.62 |
| S2 | 1.38 | (-0.69 , 3.46) | 0.19 |  | 1.48 | (-0.55 , 3.51) | 0.15 |
| T1 | 0.28 | (-0.79 , 1.34) | 0.61 |  | 0.68 | (-1.39 , 2.75) | 0.52 |
| T2 | 1.18 | (-1.16 , 3.52) | 0.32 |  | 0.59 | (-2.09 , 3.26) | 0.67 |
| I1 | 2.17 | (0.18 , 4.16) | 0.03* |  | 0.21 | (-0.77 , 1.19) | 0.67 |
| I2 | 1.08 | (-1.54 , 3.70) | 0.42 |  | 1.50 | (-0.90 , 3.90) | 0.22 |

ETDRS, Early Treatment Diabetic Retinopathy Study; β, Regression coefficients; CI, Confidence interval; F, fovea; S1, superior segment 1; N1, nasal segment 1; I1, inferior segment 1; T1, temporal segment 1.*significant values. Adjustment for age, mean arterial blood pressure, diabetes status, low-density lipoprotein, body mass index, and sex.
